# Cellulose: A Review of Water Interactions, Applications in Composites, and Water Treatment

**DOI:** 10.1021/acs.chemrev.2c00477

**Published:** 2023-01-09

**Authors:** Anita Etale, Amaka J. Onyianta, Simon R. Turner, Stephen J. Eichhorn

**Affiliations:** † Bristol Composites Institute, School of Civil, Aerospace and Mechanical Engineering, University of Bristol, University Walk, BristolBS8 1TR, United Kingdom; ‡ School of Biological Science, University of Manchester, Oxford Road, ManchesterM13 9PT, U.K.

## Abstract

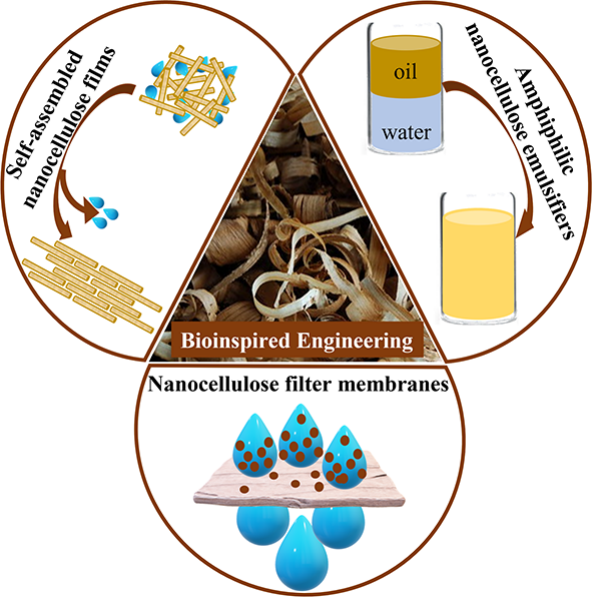

Cellulose is known to interact well with water, but is insoluble in it. Many polysaccharides such as cellulose are known to have significant hydrogen bond networks joining the molecular chains, and yet they are recalcitrant to aqueous solvents. This review charts the interaction of cellulose with water but with emphasis on the formation of both natural and synthetic fiber composites. Covering studies concerning the interaction of water with wood, the biosynthesis of cellulose in the cell wall, to its dispersion in aqueous suspensions and ultimately in water filtration and fiber-based composite materials this review explores water–cellulose interactions and how they can be exploited for synthetic and natural composites. The suggestion that cellulose is amphiphilic is critically reviewed, with relevance to its processing. Building on this, progress made in using various charged and modified forms of nanocellulose to stabilize oil–water emulsions is addressed. The role of water in the aqueous formation of chiral nematic liquid crystals, and subsequently when dried into composite films is covered. The review will also address the use of cellulose as an aid to water filtration as one area where interactions can be used effectively to prosper human life.

## 1 Introduction and Background

**Figure 1:**
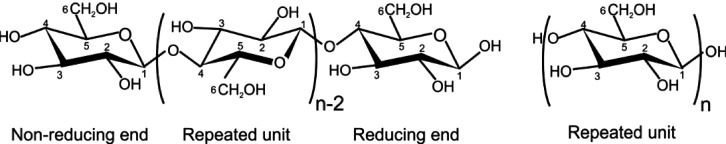
Polymeric structure of cellulose. The left-hand structure shows the nonreducing and reducing ends of the chain, and the right the repeat unit (anhydroglucose). Reproduced with permission from ref (2). Copyright 2017 Springer-Nature.

Polysaccharides cover a broad range of sugar-based polymeric materials that are the structural basis for plants, mycelium, and some animalia. Cellulose is one such polysaccharide that covers this broad range of life on earth and is the most abundant material on earth.^1^ Cellulose is an unbranched homopolysaccharide, which comprises long chains of β-d-glucopyranose joined by β(1→4) glycosidic bonds (Figure 1).^1^ The chains of cellulose have a nonreducing and a reducing end. The repeat unit, or monomer to use a polymer synthesis term, of cellulose is glucose and not cellobiose as some have reported.^2^ While the monomer and cellobiose are soluble in water, cellulose is remarkably recalcitrant to a number of solvents including water.^3^ The insolubility of cellulose is often attributed to the extensive hydrogen bonding present in the crystalline regions of the material,^4^ although not exclusively, because so-called “amorphous” regions may too contain hydrogen bonding but presumably not to the same extent, and amorphous cellulose is also insoluble to water. This adds further complexity to the issue of solubility, and the interactions that are responsible for the recalcitrance of the material to aqueous solvents not just water. Recently the role and importance of hydrogen bonding’s contribution to the recalcitrance of cellulose to solvents has been critiqued, and other nonbonded interactions have been emphasized as being just as important, if not more so.^5^

Indeed, much has in recent times been made about the importance of hydrophobic interactions in cellulose, viewing the molecule as “amphiphilic”, with the Lindman hypothesis stating that this is a major reason why cellulose is recalcitrant to most solvents, including water itself.^6^ It is paradoxical though that modified celluloses, such as methyl cellulose, are soluble in water up to ∼55 °C, and yet they would be expected to have strong hydrophobic interactions.^7^ This solubility presumably occurs due to the interruption of hydrogen bonding because of the substitution of hydroxyl groups, but again does not completely explain the paradox. Recently, “sweet spots” have been identified in the modification of cellulose, making it possible to water process derivatives into fibers and other structures^8^ in a way that could be used as a means to move away from traditional melting of polymers, demonstrated for silk but applicable to other biopolymers.^9^ Despite these recent advances, there is still much to be understood about how the duality of the cellulose molecule (hydrophobic/hydrophilic) plays out in terms of its assembly and how it can be exploited to produce new materials. To dismiss hydrogen bonding completely seems counter to what might occur in the assembly, whereby these nonbonded interactions give rise to favorable conformations of the chains to facilitate other interactions, e.g., hydrophobic. Much research has been carried out into the self-assembly of synthetic amphiphilic block copolymers, and their use for a number of applications including the synthesis of drugs and gene delivery,^10^ ordered polymer matrices,^11^ and for a range of products including rheological modifiers, stabilizers for latexes, etc.^12^ Similar studies have been conducted on incorporating polysaccharides into what are called glycopolymers, where synthetic polymers are decorated with pendant sugars, or polysaccharides are modified with synthetic polymers.^13^ Some merging of these disciplines is however required, and a deeper understanding of the interplay of side chain modification in the solubility and then perhaps switching to an insoluble state needed. The definition of what constitutes a glycopolymer is also somewhat confused^13^ and perhaps should only refer to a synthetic polymer with pendant carbohydrates or sugars.^14^ Probably most pertinent to this review will be what are called amphiphilic glycopolymers (AGPs) where hydrophilic polysaccharides, or sugars, are modified with hydrophobic groups.^13^ It may be possible that such “glycopolymers” form in the cell wall, during synthesis, but this remains a topic for future work.

Cellulose is known to interact well with water, given the large number of hydroxyl groups along its molecular chains. Cellulosic materials swell and will disperse in water. These interactions have been used as a processing and activation step in the dissolution in other solvents, and the medium provides the means for the dispersion of fibers to produce paper. However, it has been shown that water acts as an antisolvent, and much better dissolution is obtained in its absence for difficult to dissolve (highly crystalline) celluloses.^15^ So it is quite possible that water interrupts the ability of certain solvents to act on cellulose and can be used as a quench for the dissolution or as a coagulating agent, for instance in the ionic liquid spinning of cellulose fibers.^16^

The swelling of cellulose in water is thought to take place due to the presence of water molecules “packing” into the disordered regions of the semicrystalline structure. Understanding of the interactions of cellulose with water began with observations of materials such as wood.^17−21^ These early observations showed that there was a hysteresis between the water adsorbed and desorbed from the structure, which was then understood on the basis that wood behaved like a swelling gel.^21^ Gels themselves are materials that have received a lot of attention in recent times, especially where polysaccharides like cellulose are concerned. The use of nanocelluloses for the production of gels has recently been reviewed.^22^ However, it is probably not a good direct comparison to compare wood and gels because orders of magnitude differences exist between the modulus and strengths of these two materials.

Cellulose based natural fibers are numerous as sources of biomass on the planet. They come in a variety of forms, depending on the plant source, e.g., flax, hemp, jute, ramie, sisal, kapok, cotton, bamboo, and miscanthus. Geographically, certain plant fibers are enormously important to the economy of specific countries. For instance, jute in India and Bangladesh accounts for the vast majority of the world’s production and has in the past significantly contributed to their own economies.^23^ Also, plant fibers have played a significant role in traditional applications of biomass, including for the construction of ropes, sails, and also paper. In the 1930s and 1940s, both in the U.S. and Europe, dwindling supplies of traditional materials led pioneers such as Henry Ford, George Washington Carver,^24^ and Norman de Bruyne^25^ to incorporate natural fibers in automotive and aerospace applications. It is known that natural fibers are susceptible to moisture, showing typical sorption/desorption hysteresis behavior,^26^ which can limit their use in certain applications. As such, there have been moves to use more highly crystalline cellulose materials, with the premise that a reduction in the so-called “amorphous fraction” of the materials might result in less susceptibility to these effects.

To address these issues, among others, cellulose nanomaterials (CNMs), in particular cellulose nanofibrils (CNFs) and cellulose nanocrystals (CNCs), have been extensively researched and reported on extensively over the past decade.^27−31^ CNFs are typically produced through the mechanical and/or chemical/enzymatic breakdown of plant matter, using processes such as homogenization, grinding, and microfluidisation.^31,32^ Similar nanofibrils of cellulose can also be made using bacteria, so-called bacterial cellulose (BC).^33^ All of these forms of CNMs have been used to make composite materials, where the high stiffness of CNFs and CNCs, due to the intrinsically high modulus of crystalline cellulose (130–150 GPa),^34^ enables reinforcement of polymer matrices.^35^ In addition, given the switchable interactions (“on/off”) between cellulose nanomaterials by the introduction and extraction of water, adaptable properties actuating stiffness upon drying, and flexibility on wetting, can be achieved.^36,37^ Indeed, nature has synthesized a complex composite system with high strength and flexibility in wood and plants, which can be changed by the addition of water. Moreover, the actual synthesis of plant cell walls requires the presence of water. This review will start by exploring the biosynthetic process of cellulose and its interaction with other components of the plant cell walls, emphasizing the role of water, and then explore extracted nanomaterials and their interactions with water in composite systems and assemblies.

## 2 Cellulose Biosynthesis and Interactions with Cell Wall Components

### 2.1 Formation of Cellulose in the Cell Wall

**Figure 2:**
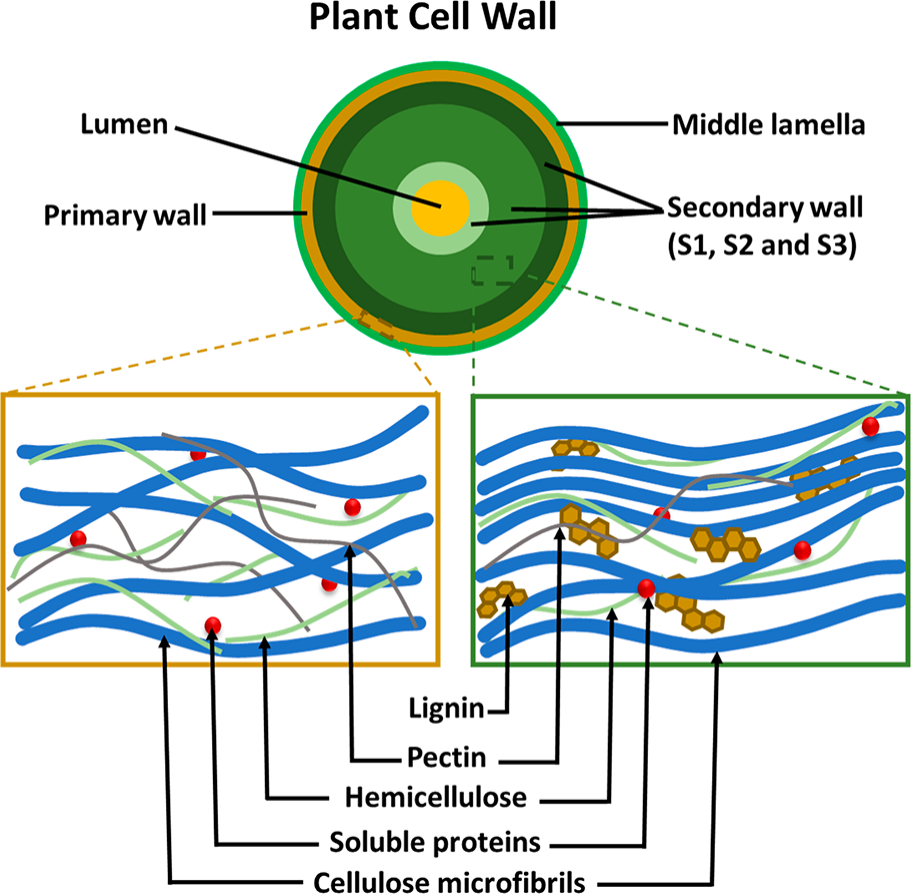
Schematic representation of the plant cell wall showing the various components that make up the primary and secondary cell walls.^39,44,45^

The growth of plants occurs via enlargement and differentiation of cells enclosed within polysaccharide-based walls.^38−40^ The cell walls of plants are divided into the primary cell wall (PCW) and secondary cell walls (SCW). The components of the PCW, which include cellulose, hemicellulose, and pectin function in a cohesive manner to enable the enlargement of plants while maintaining their structural integrity.^38,41−43^ The SCW is deposited after cell growth has stopped and contains a greater portion of cellulose alongside hemicellulose, lignin, and a smaller amount of pectin.^39,44,45^ The plant cell wall and the major components of the PCW and SCW are represented in Figure 2.

Cellulose biosynthesis in the cell wall of plants is a highly complex and concerted sequence of natural processes. However, advances in gene sequencing have paved the way for a better understating of cellulose synthesis in the cell walls.^44,46^ The biosynthesis of cellulose occurs at the plasma membrane of plant cells by a large mobile enzyme complex known as the cellulose synthase complex or rosette.^38,39^

**Figure 3:**
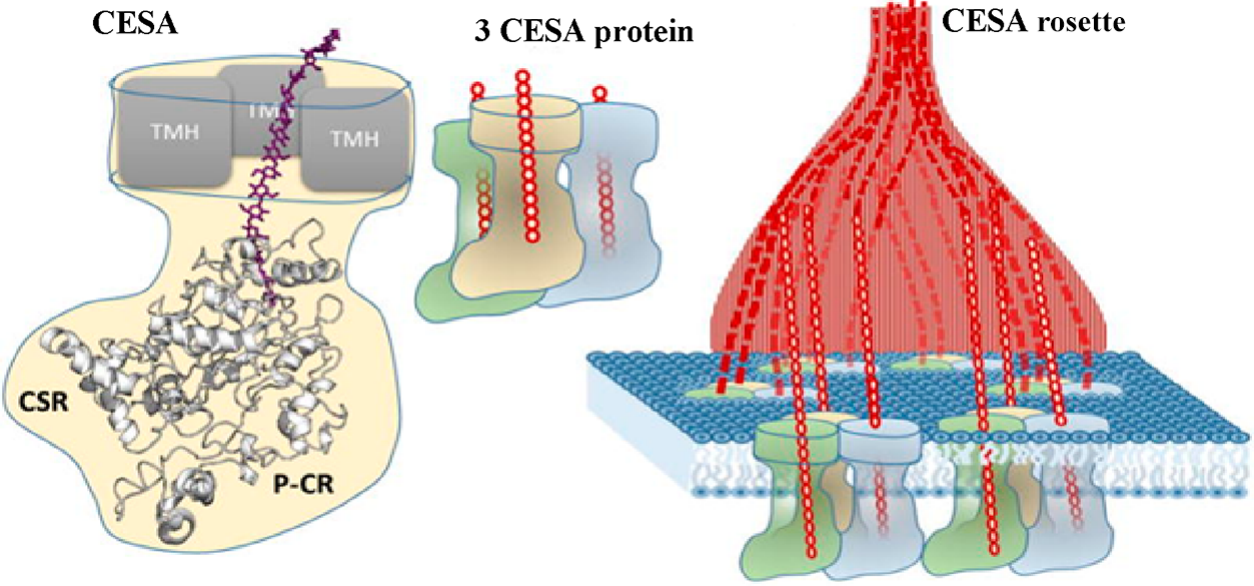
Representation of the 18 chain model of cellulose synthase protein composed of 6 trimers. Reproduced with permission from ref (42). Copyright 2014 Elsevier Ltd.

The catalytic subunits of the CSC are known as CESA proteins. The model plant *Arabidopsis thaliana* possesses 10 different CESA proteins, and three different CESA proteins are needed to make a functional cellulose synthase complex. *AtCESA*1, 3, and 6 are involved in cellulose biosynthesis in the PCW, while *AtCESA*4, 7, and 8 are responsible for the synthesis of cellulose in the SCW. Each CESA protein uses uridine diphosphate (UDP)-glucose as substrate to catalyze the addition of β-d-glucopyranose to the growing cellulose chain via a (1→4) glycosidic bond.^41,46,47^ While early studies have suggested the basic cellulose microfibril is composed of 36 chains,^38^ more recent work using wide-angle X-ray scattering and solid-state NMR^48,49^ all suggests an 18–24 glucan chain cellulose microfibril^42,48,50^ An 18-glucan chain model^51^ was further supported by several studies on CESA protein structure and organization, including a recent study using cryoelectron microscopy looking at poplar CESA8, part of the cellulose synthase enzymes in the SCW of poplar.^52^ The structure demonstrated that PttCESA8 formed a homotrimer that would be consistent with a rosette synthesizing an 18 chain microfibril and being composed of 6 trimers (Figure 3).^42,53^ While several reports also suggest a rosette composed of 6 CESA trimers, some evidence supports CESA dimerization.^54−56^ This includes the recent crystal structure using the catalytic domain of CESA3 that was solved as a dimer.^54^ It is hard to reconcile a dimer with 18 chain microfibril, one suggestion is that dimers may be some sort of assembly intermediate that form prior to assembly into an entire rosette.^54,55^

**Figure 4:**
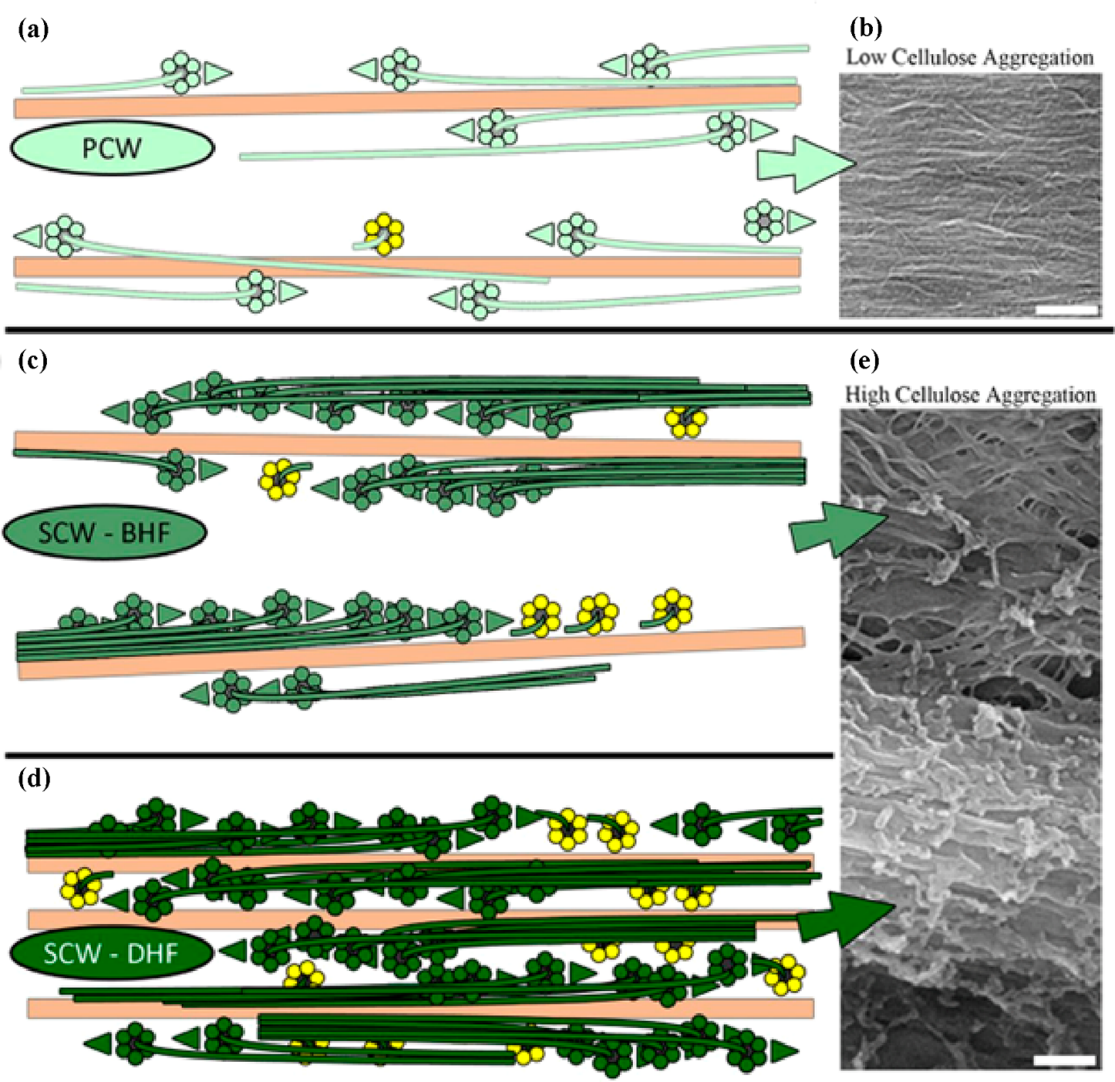
Models and scanning electron microscope images depicting the aggregation of cellulose microfibrils in PCW (a) and (b) and in SCW before early formation (BHF) (c) and (e) and during late formation (DHF) (d) and (e); scale bars = 200 nm. Reproduced with permission from ref (44) (CC-BY).

Polymerization of the glucan chains by the rosette results in the formation of cellulose microfibrils in both PCW and SCW.^41^ The products from individual rosettes may further aggregate to form larger microfibrils. An inducible xylem vessel transdifferentiation system has been used to study the relationship between the cellulose synthesizing rosettes and microfibril structure. The system can produce localized SCWs that are similar to native cell walls but is also amenable for live imaging of cellulose synthase complexes.^44^ This study showed that there is increased aggregation and bundling of cellulose microfibrils in the SCW in comparison to the microfibrils in the PCWs as shown in Figure 4. This aggregation results from an initial even and directional distribution of the cellulose synthase enzymes in the plasma membrane during SCW synthesis that subsequently work in a concerted fashion during SCW synthesis to form larger aggregates.

The directional confinements of the cellulose synthase complexes are perceived to be arising from possible physical attractions between the complexes within the plasma membrane, aided by other components of the SCW, such as hemicellulose and lignin. These aggregated fibrils were however present in both discrete lignin containing and nonlignin containing thickenings of SCW.^44^

### 2.2 Interactions between Cellulose and Other Cell Wall Components

The cell walls of plants are complex composite systems. Interactions between cellulose and other cell wall components (hemicellulose, lignin, pectin, and water) occur via hydrogen bonding, van der Waals forces, and electrostatic and hydrophobic interactions.^57^ Understanding these interactions would be very crucial for the development and engineering of cellulose-based composite materials that are fit for purpose.^58^ Furthermore, an understanding of the role that water plays in the interactions between these components is needed.

In plant cells, the matrix polysaccharides, hemicellulose, and pectin, are synthesized in the Golgi apparatus and transported to the cell wall.^38^ Hemicellulose is a branched polymer of different sugar units that binds well to cellulose in the cell wall.^59^ Pectin, a complex polysaccharide, supports cell growth by forming swollen gels that cause microfibrils to glide past each other during growth or lock them after growth.^60^ There is evidence that some types of hemicellulose can bind to cellulose in a manner that they are able to alter the packing and the crystal structure of cellulose microfibrils.^61,62^ Cultures of *Acetobacter xylinum* prepared in acetyl glucomannan^61^ and xylan^62^ hemicellulose media resulted in loosening of the microfibril packing and a reduction in the cellulose 1α content because of the binding of these hemicelluloses. It is worth noting that on the contrary, cellulose microfibrils prepared in pectin media did not affect cellulose packing or crystalline structure.

Some postulations of cellulose–hemicellulose interactions in the PCW may involve a spontaneous binding of neighboring microfibril bundles by hemicellulose or the entrapment of hemicellulose during microfibril formation and subsequent coating by the same.^38^ The coating of cellulose microfibrils with matrix polysaccharides functions as a “glue” for the nearby microfibril bundles. In a recent study,^63^ the interaction of cellulose with five hemicelluloses (galactoglucomannan, *O*-acetyl-galactoglucomannan, 4-*O*-methylglucuronoxylan, 4-*O*-methylglucuronoarabinoxylan, and fucogalactoxyloglucan) found in PCW and SCW were studied using molecular dynamics simulations in hydrated and nonhydrated systems and in isolation of other cell wall components. This study showed that 4-*O*-methylglucuronoarabinoxylan has the highest binding energy to the hydrophilic plane of cellulose, while fucogalactoxyloglucan showed the least binding energy. A binding assay study of cellulose–xyloglucan interaction however showed that the binding capacity of xyloglucan depends on the molecular weight, as lower molecular weight xyloglucan resulted in higher binding capacity than those of higher molecular weight.^64^ This study also postulated that the interaction of xyloglucan with hydrated cellulose involves the formation and breakage of hydrogen bonds.^64^ Nevertheless, the binding capacity of either xyloglucan or xylan depends on the source of cellulose, whether of bacterial or plant origin and the differences in packing within the cell wall.^65^

**Figure 5:**
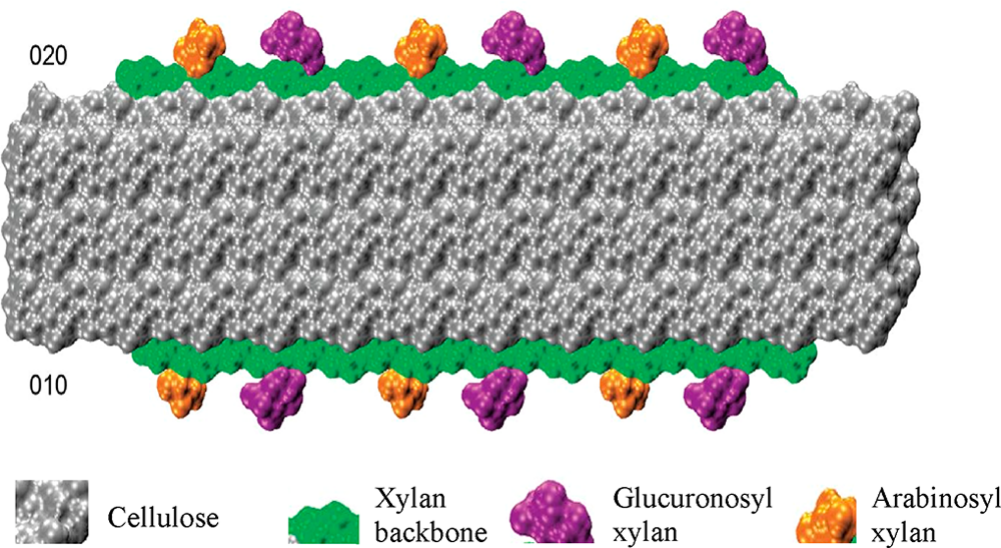
Interaction of glucuronosyl and arabinosyl substituted xylan on the equivalent hydrophilic (020) and (010) surfaces of cellulose, exposing the hydrophobic plane to possible interactions with lignin in the SCW. Adapted with permission from ref (66) (CC-BY image).

According to Busse-Wicher et al.^50,66^ a well-defined xylan interaction with the hydrophilic plane of cellulose within the secondary cell wall is possible if the xylan chains conformed to a 2-fold helical screw, such as seen for cellulose, where only one side of the xylan chain is bearing well distributed acetyl or [4-*O*-methyl] glucuronic acid functional groups. However, irregular binding of xylan to the hydrophobic planes of cellulose was postulated to occur. The authors also asserted that this concerted covering of the hydrophilic plane of cellulose by xylan exposes the hydrophobic planes of cellulose to interaction with lignin as represented in Figure 5.^66^

The 2-fold helical screw conformation postulated for xylan by Busse-Wicher et al.^50,66^ was later confirmed to facilitate xylan interaction with cellulose, as evidenced by solid-state nuclear magnetic resonance (ssNMR) studies using whole/untreated plant stems.^67^ However, this study shows that xylan acts as a bridge between the cellulose and lignin and that the cellulose–lignin interaction is limited. Also, it was indicated that the xylan–lignin interaction occurs via intermolecular hydrogen bonding between the oxygen of the lignin methoxy groups and the hydrogen of the hydroxyl groups of xylan^67,68^ and very minimally by a hydrophobic interaction.^67^ Molecular dynamics simulation of the interaction of lignin with cellulose, however, showed higher lignin binding affinity on the hydrophobic plane of cellulose.^69^ It is therefore likely that lignin interacts with cellulose and other cell wall components via both electrostatic and hydrophobic interactions, which is something that could be potentially mimicked in synthetic systems. There is clearly much more work to do, however, to ascertain which interaction is dominant and the mechanism by which cellulose interacts with xylan and lignin in the cell wall.

Indeed, lignin is a hydrophobic aromatic polymer which is predominant in the secondary cell wall of plants and woody materials, and contributes to the mechanical and waterproofing properties of these materials.^67,68^ In an attempt to mimic the cell wall of wood, honey-combed cellulose films were fabricated and adsorbed with hemicellulose and/or lignin.^70,71^ The films with adsorbed lignin showed improved mechanical strength even at high moisture content in comparison with films without lignin at the same conditions.

Water is actually a major cell wall component that regulates the growth of plants but is less talked about relative to the solid and polymeric structural constituents.^59,72^ Water uptake supports the growth of plants by increasing the volume and relaxing the stress generated in the cell wall from the synthesis of cell wall polymers.^73,74^ Water is therefore an essential component of the complex cell wall composite that interacts with the other cell wall components at different rates.^75^

In simulated cellulose–hemicellulose interactions, water was implicated to cause a reduction in the amount of force needed to shear the cellulose–hemicellulose system by acting as lubricant and plasticizer within the interface.^63,76^ Another molecular dynamics simulation of cellulose–hemicellulose interaction showed that water molecules were not adsorbed within the crystalline regions but adsorbed on hemicellulose and the interphase between the two polymers, leading to increased spacing and a reduction in modulus.^77^ Simulations that did not include hemicellulose, but only cellulose and water, showed that there is an increase in the volume of the crystalline region of cellulose on interaction with water.^57^ This model, however, may not be representative of the cell wall as it is devoid of other cell wall components.

One stark observation is the disparities in the literature on the understanding of cellulose formation in the cell wall and the interactions of cell wall components. This lack of agreement stems from the great diversity and heterogeneity of the material in question,^78^ cellulose, the most abundant material on earth. While this lack of consensus can appear challenging with many begging questions yet to be answered, there lies a great opportunity to harness the manifold knowledge and understanding of cellulose formation and interactions to create sustainable materials for specific applications. There have been many attempts to harness the self-assembling nature of cellulose, and this review now turns to a few examples of those trying to understand the role that water plays in these processes.

## 3 Self-Assembly of Chiral Nematic Phases in Aqueous Systems

### 3.1 Chiral and Twisted Shapes of Cellulose and Water Interactions

**Figure 6:**
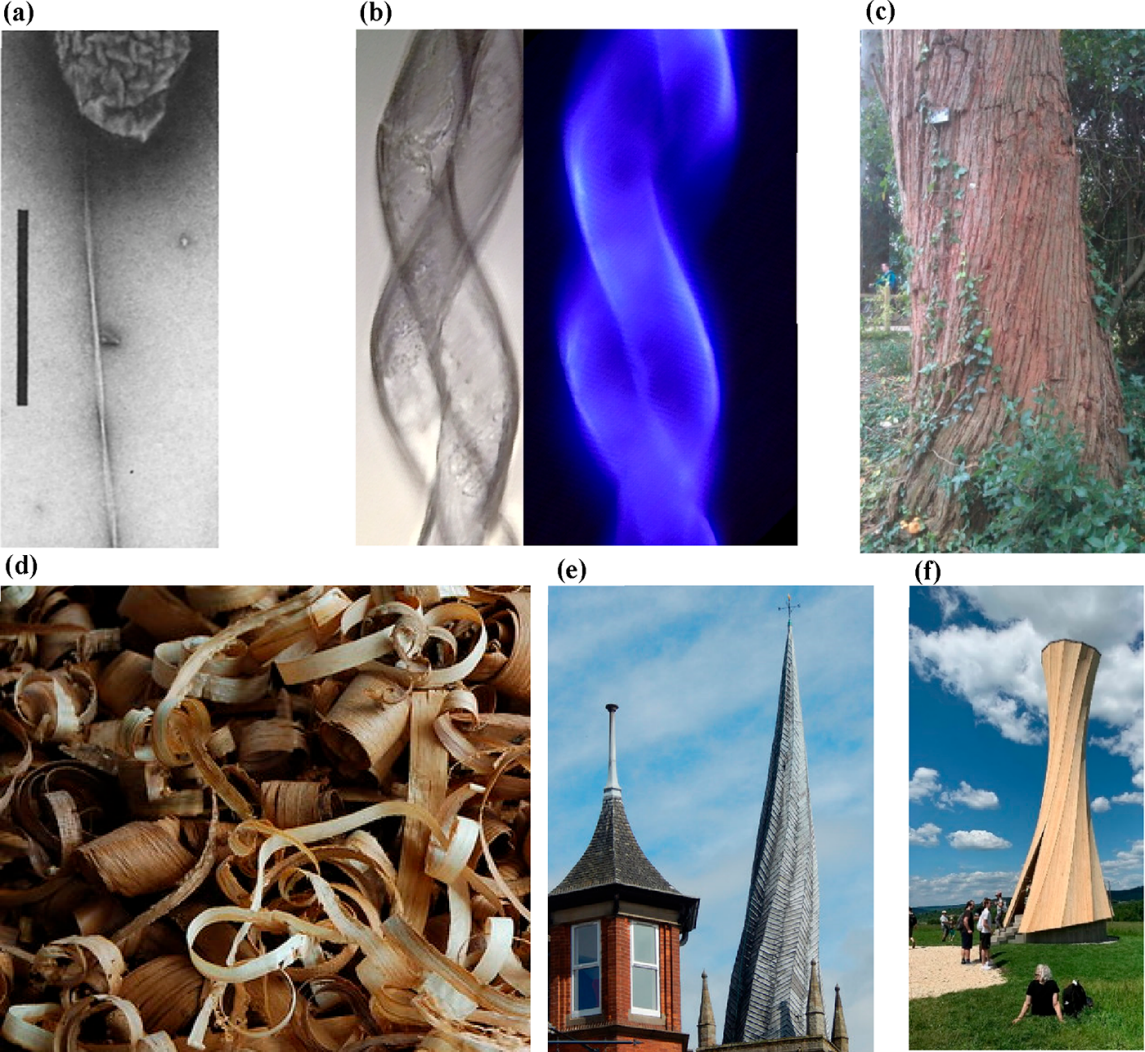
Chirality of cellulose and cellulosic materials. (a) Chiral twisting of a single fibril of bacterial cellulose (image has been rotated for clarity, and scale bar = 1 μm).^84^ (b) Twisting of cotton fibers (image courtesy of R. M. Brown). (c) Image of a twist in the trunk of *Eucalyptus gigantea* (photograph taken in the grounds of Exeter University (S .J. Eichhorn)), (d) Twisted wood shavings (CC-BY image). (e) The twisted spire of Chesterfield church, UK (CC-BY image), and (f) the Urbach tower in Baden-Württemberg, Germany (CC-BY image).

It has been known for some time that cellulose possesses a chirality, not least at the molecular level, but also at other length scales.^79^ Cellulose itself forms into many chiral shapes, including at the fibril level (see Figure 6a for a twist in a bacterial cellulose fibril) and the macroscale in plants (see Figure 6b,c for twists in cotton fibers and the trunk of a tree). Other chiral forms of cellulose, such as the twists of wood shavings^80^ (Figure 6d), rely on the inherent twist of the microfibrils in the S2 layer of the cell wall. It is intriguing how this twist can also be controlled by the presence of water. Interesting twisting of wooden structures also occur in situ, for instance, the twisted spire in the UK town of Chesterfield (Figure 6e), thought to be due to a combination of the warping of the lead coating in the sun but also facilitated by the twisting of unseasoned (“undried”) wood used for its construction. Twists in wood can be made to occur, such as for the Orbach tower in Germany (Figure 6f), which is made from laminated wood that has been cut at different angles to the grain direction. This approach has recently been demonstrated to generate bending in wood, through a change in the moisture content upon drying,^81^ although such effects have been known about for some time. These larger scale twists, however, can exhibit themselves in either left- or right-handedness, whereas at the molecular scale, and indeed levels just above this chiral handedness is very specific; in chiral nematic structures, only left-handed helices are observed.^82,83^ So relationships between different types of chirality along the length scales are unfounded, moreover, the relationship between chirality and water is also not well-understood, even if there is one at all. While the interaction of moisture with wood is better understood, its role at the molecular scale is less well articulated. It is to this topic that we will now turn and in discussing chiral nematic structures and the possible role of water in their formation.

### 3.2 Chiral Nematic Liquid Crystalline States of Cellulose

**Figure 7:**
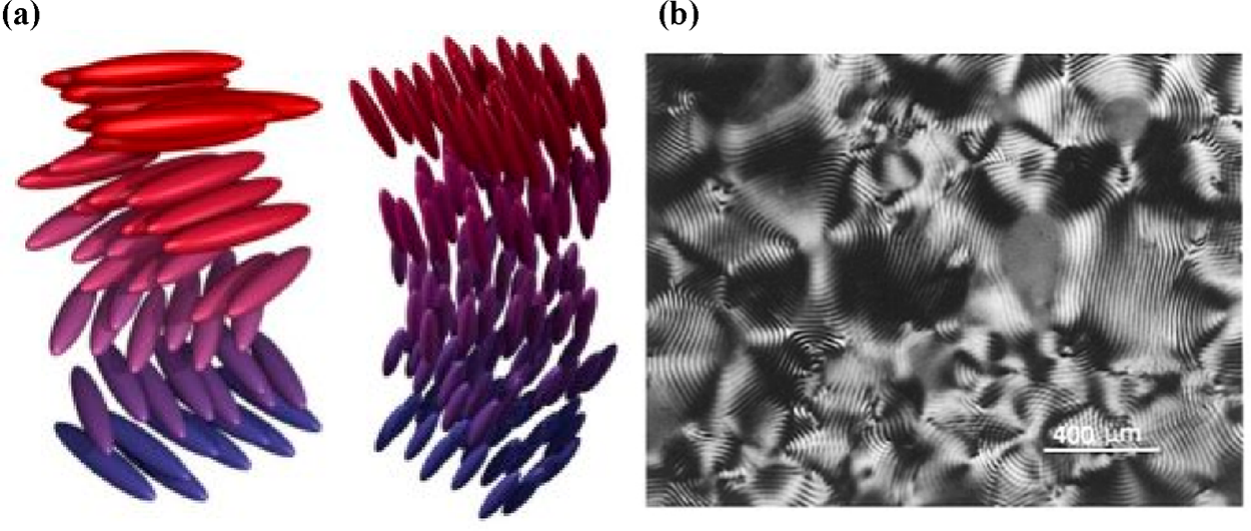
Typical chiral nematic phases for rod-like molecules in aqueous solutions that form spontaneously. (a) The left-hand helical phases are found for CNCs, but the right-hand chiral phases are precluded. (b) A typical “fingerprint” texture of a chiral nematic phase within the anisotropic region of a suspension of CNCs viewed under cross-polarized light. Reproduced with permission from ref (102). Copyright 1996 American Chemical Society. Image provided courtesy of Prof. Derek G. Gray (McGill University, Canada).

One of the very interesting and intriguing chiral structures that cellulose nanofibers can form is that of a liquid crystalline cholesteric, or what is also termed a chiral nematic phase.^85^ Cholesteric liquid crystalline (LC) structures for a variety of biopolymers have been known about since the early 1800s and has been observed in aqueous solutions of DNA,^86−90^ polypeptides,^91−93^ cellulose,^94,95^ virus suspensions,^96,97^ helical filaments,^98^ and amyloid fibrils.^99^ We know from all of these studies that LC phases are often formed due to the presence of charged rod-like particles in an aqueous phase.^100^ Indeed, although an aqueous environment is often used, this effect will occur in other solvents. A combination of and a balance between chiral steric interactions and Coulombic charge repulsions, often CNCs that form LC phases are negatively charged with sulfate half ester groups, is key to the formation of the lyotropic phases.^100^ CNCs in a dilute aqueous suspension are known to form isotropic fluids, but at a very specific concentration they will form LC phases.^101^ The specific nature of these LC phases are chiral nematics, wherein CNCs are ordered nematically, but a continuous twist is observed (Figure 7a) along a vertical axis, which gives rise to interesting optical properties (Figure 7b), wherein circularly polarized light is reflected from such structures with an iridescence.

The exact physical reason why CNCs form this phase is unknown, nor is it known what role water plays in this transition to a chiral phase. What is known though is that colloidal stability, often for CNCs controlled by the presence of sulfate monoester groups on their surface, is extremely important.^102^ It has been shown that the electrostatic repulsion between rods decreases the concentration at which the chiral nematic (or ordered) phases occur, to a value below that which causes purely geometry driven kinetic arrest (or the formation of a gel).^102^

**Figure 8:**
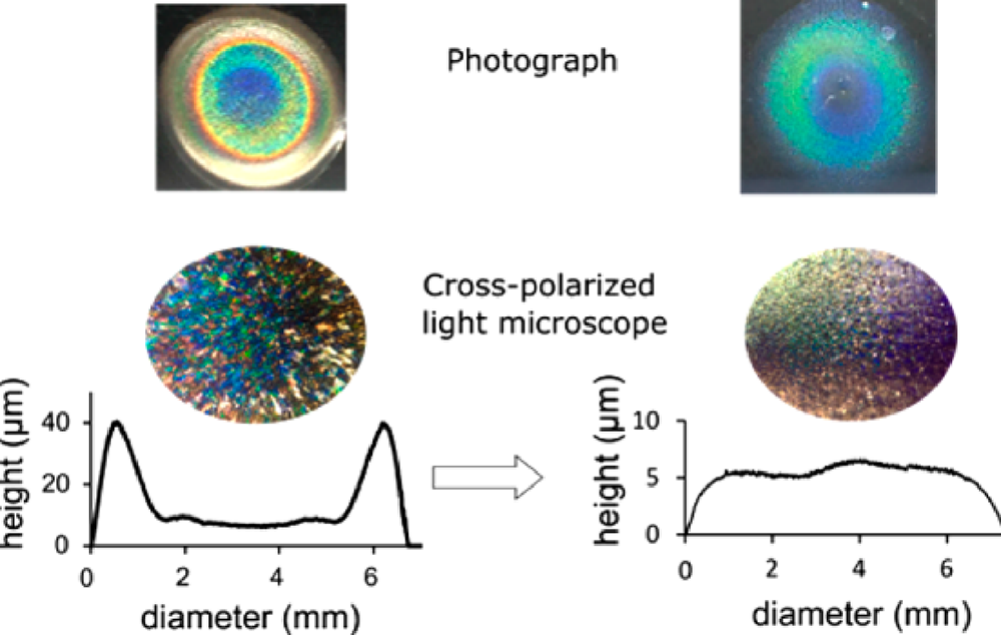
Photographs, crossed polarized light micrograph images, and height profiles of drying droplets of CNC films containing chiral nematic structures. (left) A film dried without the presence of ethanol, and (right) a film dried with a 60:40 v/v ratio of ethanol:water. Reproduced with permission from ref (109). Copyright 2017 American Chemical Society.

Very few studies have specifically looked at the CNC–water interactions with respect to this self-assembly process. One area where this has been better understood though is in the drying of the liquid crystalline state of CNCs into solid films, so in the absence or reduction in the aqueous solvent state. The formation of structurally colored films of CNCs by controlled drying of the liquid and chiral crystalline state has been known for some time.^103^ The approach has recently been a subject of renewed interest due to the ability then to take such structures and covert them to silica, and glass-like materials,^104^ but to also scale the production to make colored films and iridescent glitters.^105^ The formation of the films involves controlled drying, which initially focused on the drying of pinned droplets to a substrate,^103,106^ although such approaches have recently demonstrated that coffee ring effects can give rise to nonuniformity in both thickness and structural color.^107^ The coffee ring effect has been known about for some time for other aqueous suspensions of nanomaterials.^108^ What is most interesting here though in a drying film of CNCs is the movement or flow of the water and how that can be controlled to enable the better formation of the resultant films. Later, the drying kinetics of the film are also important. It is known that Marangoni and capillary flows are in competition with each other in a drying droplet of aqueous CNC suspensions.^109^ The Marangoni effect has long been known and is the mass transfer along the interface between two fluids due to a gradient in the surface tension, and it is known that it has to be suppressed in a drying droplet for coffee rings to form.^110^ The manipulation of this flow has been studied for CNC based aqueous droplets and shown to be insignificant compared to the capillary flow.^109^ If an infusion of ethanol into the droplet is however allowed, then Marangoni flows can be used to ensure the production of uniform films of structural color.^109^ Height profiles of the drying droplets have been shown to be more uniform (see Figure 8) for samples containing a 60:40 v/v ratio of ethanol to water.

It is clear from these images in Figure 8 that a more unform thickness film was produced with a commensurate structural color. These flows have subsequently been shown to be important for coalescing tactoids within drying droplets.^111^ Tactoids are discrete ordered “droplets” of a liquid crystalline phase, within a more disordered and isotropic phase, in this case the main droplet of liquid and solid phase of CNCs.^111^ Furthermore, the hydrophilicity/hydrophobicity of the surface on which the main droplets form has an effect on the both the flow of the aqueous phase and thereby the tactoids within the main droplet.^111^

**Figure 9:**
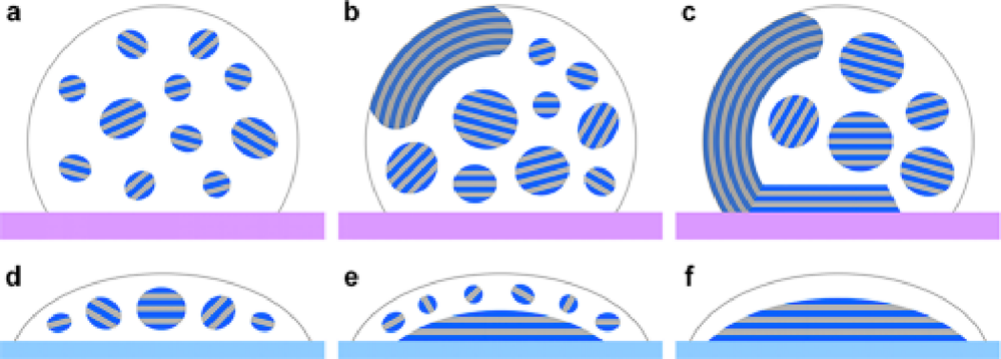
Schematic showing the development of discrete liquid crystalline tactoids and continuous chiral nematic phases in aqueous CNC droplets formed on (a–c) hydrophobic PTFE and (d–f) hydrophilic glass substrates. Folded disclinations of coalesced tactoids form for (a–c), whereas more uniform and base deposited films form for (d–f). Reproduced with permission from ref (111). Copyright 2019 American Chemical Society.

Hydrophobic surfaces lead to the formation of disclinations where coalescing tactoids are forced toward the edge of the drying droplet, whereas a hydrophilic material gives rise to deposition from the base of the droplet (Figure 9).^111^

This control of the flow of water is just one aspect of the formation of the entire film of a deposited cellulosic structure. There have been very few studies on the role of water in the interstices between the CNCs in a forming chiral nematic structure. It is known that because CNCs are themselves parallelepiped structures, with twists of their own, that they pack in a chiral structure to maintain efficiency.^112^ Recently, hard-particle models of CNCs self-assembling in aqueous and apolar suspensions have been reported, showing an entropic effect of assembly, although the role of water in this was not explicitly described.^112^ It would be interesting to see if there is an entropic gain from a displacement of water molecules between two adjoining CNCs in a modeling situation, but this would require explicit water molecules to be simulated. It is known that highly hydrophobic CNCs (modified using octylamine groups) do not form chiral nematic structures due to rapid gelation,^113^ and this is due to the hydrophobic interactions between the CNCs. However, little has been reported on any such effects in unmodified CNC systems or indeed whether such effects do occur. Some understanding of the shape of confined water around CNCs, with hydrophilic and hydrophobic moieties present, has recently been described.^114^ The drying rates of CNC films has been controlled by the presence of hydrophobic groups (phosphonium modification).^114^ This work showed that the dielectric constant of absorbed water is significantly lower than that of the bulk, and the shape of the confined layer is more spherical for hydrophobically modified CNCs and platelet-like for hydrophilic materials.^114^ The shape of the confined layer of water then has an effect on the pitch of the cholesteric twist, and thereby the color, with hydrophilic CNCs giving rise to a tighter twist, and a red-shift in color.^114^ Although the more hydrophobic forms of CNCs and other nanocelluloses do not seem to form chiral structures, they do however aid self-assembly and offer interesting routes to structured materials. The next section deals with this topic, highlighting again the role of water in this process.

### 3.3 Hydrophobic Interactions and Amphiphilic Forms of Cellulose and Oligomers for Self-Assembly

Amphiphilic properties of charged forms of cellulose, such as sulfated cellulose nanocrystals and nanofibrils, are well-known, although sometimes overstated. It is understood that the surface of cellulose itself is relatively inactive and requires some modification to increase its chemical activity. Nevertheless, adsorption of many different polymeric and other chemical species to its surface is possible, driven by both enthalpic and entropic processes.^115^ The adsorption of chemical species to the surface of cellulose, unmodified or not, is best understood in terms of thermodynamics.^115^ It has been shown that for a wide range of materials (polymers, proteins, charged particles) that they demonstrate an invariance in the binding based on thermodynamic considerations, with a constant change in the Gibbs Free Energy (Δ*G*).^115^ Linear relationships between the enthalpy change and entropy change were plotted, showing a constant slope equal to the reference temperature (fixed in the analysis), for a wide range of different adsorbants.^115^ The mechanistic differences were that the binding of proteins was found to be enthalpy and entropy driven, whereas charged molecules and ions were found to be driven by entropy, wherein water molecules are displaced but there is no heat exchange.^115^ These considerations are important when it comes to discussions about so-called hydrophobic interactions because there are many recent publications that claim that cellulose has hydrophobic and hydrophilic properties, i.e., it is amphiphilic.^6,116,117^

In simulation studies of cellulose–graphene interfaces, it has been shown that certain faces of the cellulose molecule in idealized structures will adhere to graphene via a hydrophobic effect, wherein water is displaced between the surfaces.^118^ The hydrophobic effect, or interaction, as it should be called (it is not a bond) has been studied extensively and is probably best understood from the simple partitioning of oil–water emulsions.^119^ It has been shown that the hydrophobic effect, even in these simple systems, has subtleties in the balance of entropic and enthalpic effects as a function of temperature.^119^ Southall et al. showed that there is a big penalty for the opening of a cavity in a solvent.^119^ These “cavities” are often viewed in aqueous systems as a structuring of the water molecules around the solute. The role of the size of the solute has been recently shown to be critical in this structuring of the solvent, and the solvent also dictates the solute identity.^120^ It is known that cellulose binds to water through hydrogen bonding and also to itself by the same interaction. But there is no difference in the strength of either bond (water–water, water–cellulose), and in that sense cellulose will just as readily bind to water as it will do to itself. Indeed, a recent overview of the role of hydrogen bonding and the exaggeration of its importance has recently been presented and has shown that their strength and relevance in the cohesion of cellulose is relatively small compared to dispersion and hydrophobic effects.^5^

**Figure 10:**
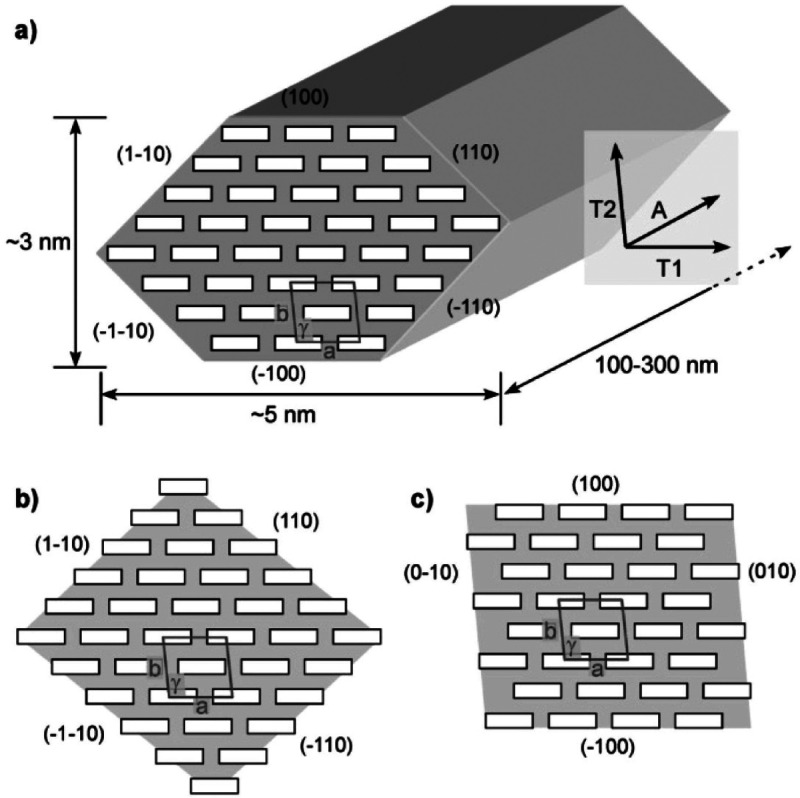
Some idealized cross sections of cellulose nanomaterials (in this case CNCs) for (a) a 36 chain model showing the dimensions of the CNC, and the various planes, with a unit cell drawn with a box with dimensions *a* = 0.786 nm, *b* = 0.817, and γ = 97°, is for a Iβ lattice, (b) an alternative representation of a 36 chain model, but this time with more hydrophilic outward facing planes (110, 1–10, −1–10, and −110), and (c) a 32 chain model with more hydrophobic planes (100, −100) exposed. A, T2, and T1 represent anisotropic axes of cellulose structures. Reproduced from ref (122), but (b) is originally the work of ref (123). Copyright 2010 American Chemical Society.

Nevertheless, what constitutes a hydrophobic face in a crystalline form of cellulose is still a subject of debate. Internally, Nishiyama has shown that almost certainly London dispersion forces are more dominant in the cohesion of crystalline cellulose than hydrogen bonding.^121^ The presence of a hydrophobic “face” to cellulose is somewhat fraught with difficulty because, for fibrillar structures, it is not completely known what their shape is and whether this varies between materials made from different starting materials. Lahiji et al.^122^ have summarized several idealized cross sections for cellulose nanocrystals (CNCs; see Figure 10). It is possible to see that there may be more hydrophobic edges, such as the 100 plane in a 36 chain model (Figure 10a), but other representations as such either diminish this possibility (Figure 10b) with more hydrophilic faces present or where there is an increase in the number of planar hydrophobic faces of the chains exposed (Figure 10c). It is also important to note that although we might talk about a hydrophobic face of cellulose, it requires another equally hydrophobic face to be in contact, because this is a dispersive effect, a hydrophobic face cannot exist in isolation. Nor can we talk of a hydrophobic face in the absence of water.

Many attempts to render nanocellulose hydrophobic have been reported, but most rely on the use of oil-based polymeric chains for this purpose. Several papers have been published that use natural chemical substances, including tannic acid to attach primary amines with long alkyl chains,^124,125^ isocyanate terminated castor oil,^126^ and fatty acids.^127^ However, all of these approaches render the cellulose completely hydrophobic, and while the materials will disperse in suitable organic solvents, they do not then disperse well in water.

**Figure 11:**
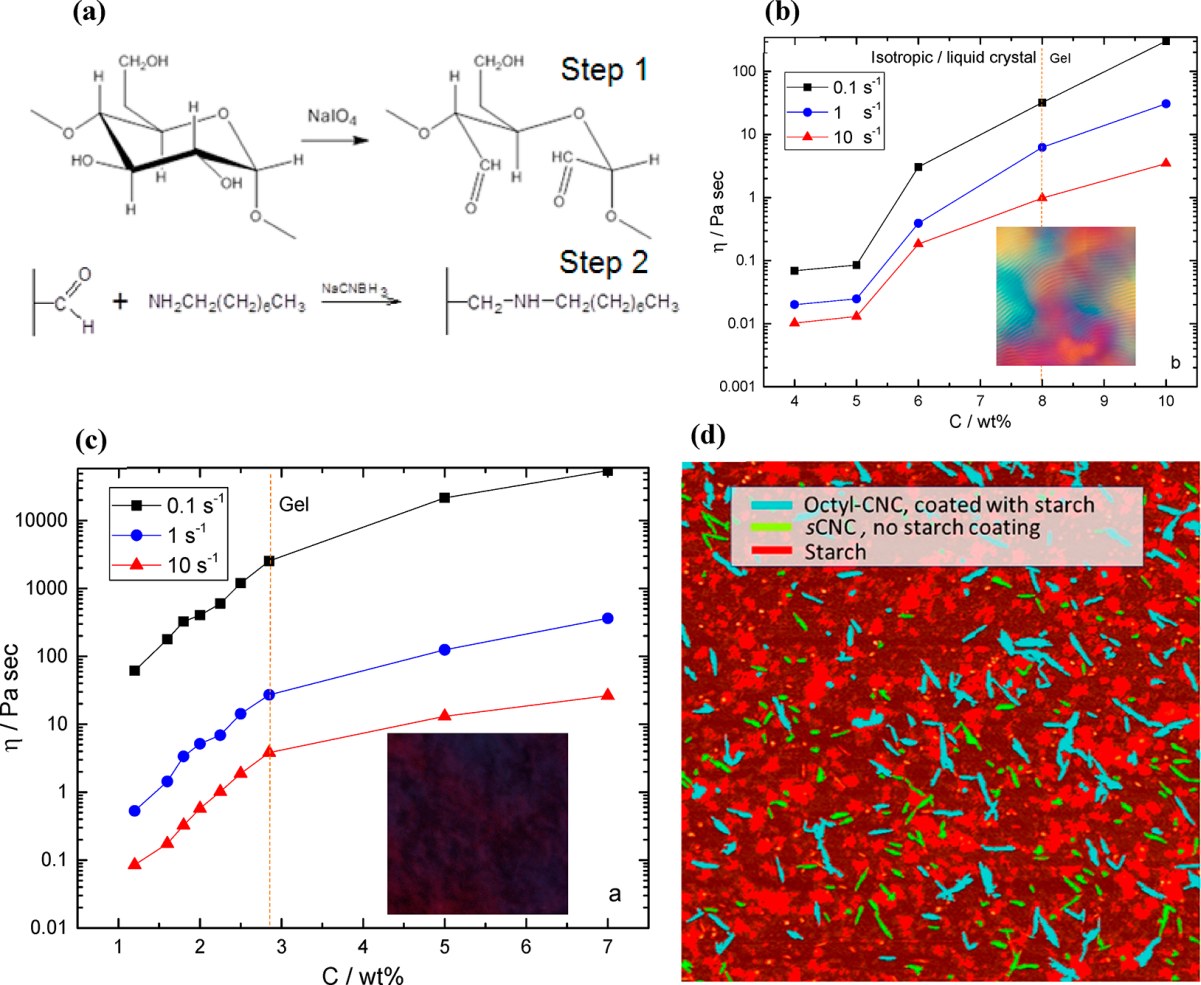
(a) Mechanism of the production of octylamine modified cellulose nanocrystals involving periodate oxidation (step 1), followed by a reductive amination of the carboxylic groups (step 2). Dependence of steady flow viscosity (η) of (b) octylamine modified CNCs and (c) sulfated CNC aqueous suspensions at three different shear rates: 0.1^s–1^, 1^s–1^, and 10^s–1^ as a function of CNC concentration (*C*). The dotted lines show the presence of the gel point, and the square insets show polarized light microscopy images of the suspensions. (d) A colored image from an atomic force microscope scan of a mixture of starch-CNC showing high contact adhesion between starch and octylamine modified CNCs (octyl-CNCs; turquoise) compared to free starch (red) and sulfated CNCs (yellow).^113^ CC-BY image.

There is this paradox of a paradigm in cellulose modification to enable interaction with hydrophobic materials that often takes the route of losing or significantly reducing its interaction with water. Some recent work has addressed this, through the modification of CNCs using octylamine groups, which while rendering hydrophobic properties to the nanomaterials, preserves the charge from the sulfate monoester groups.^113,128^ This enables these nanomaterials to be processed in water because they also continue to be dispersible in polar solvents. The chemical procedure for doing this involved a periodate oxidation of the cellulose, followed by a reductive amination of the carboxylic groups (see Figure 11a). These modified CNCs are then able to form strong gels in water. CNCs that are only sulfated were found to form gels at relatively high concentrations of the solid nanoparticle content (∼8%), and they also formed anisotropic liquid crystalline phases (see Figure 11b). When the CNCs are modified with octylamine groups, while still dispersing in water they formed strong gels at lower concentrations (<3%) and did not show any liquid crystalline phase: the samples just moved to the gel state (see Figure 11c). In addition to this, it was found that starch adhered to the octylamine modified materials more readily than the sulfated, on account of what is thought to be a hydrophobic interaction, as subsequent work has demonstrated.^128,129^ This work has recently been extended to show that octylamine modified CNCs can form much more stable Pickering emulsions than sulfated materials and thereby be used in self-healing composite varnish coatings.^130^ These modified nanocellulose forms could be used more widely in the development of structured composites that have charge. What has not been explored is the propensity for nanocellulose to continue to interact with water in its application phase. Adaptive composite materials that respond to water and actuate upon interaction have been covered in other reviews.^131,132^

One of the key applications though that exploits the interaction of cellulose with water is in filtration, and the review will now turn to that topic.

## 4 Water Responsive Nanocellulose-Based Composites

Building on the work presented in the previous section on chiral nematic films and liquid crystalline states of CNCs, switchable interfaces in chiral-based epoxy–CNC composites have been also achieved by combining cholesteric phase liquid crystalline based films with epoxy resin; a water responsive chiral nematic composite.^133^ Interactions between the CNCs were interrupted by water–OH bonding when wet, which increased the toughness (×4 times) of the films considerably, whereas when dry the films were found to be much stiffer and stronger.^133^ The ability for cellulose to “switch” from interactions with itself, to interactions with water, is grounded in the fact that a hydrogen bond within the structure of cellulose is no stronger, nor more energetically favorable, than with water.^6^ However, it is also known that there is a certain amount of “free water” at the surface of cellulose even at very low moisture contents,^134^ and that as moisture content increases this also increases, relying on the presence of bound water also at the surface. Quite what the role water plays in mediating the interactions between cellulose fibers, particularly at the nanoscale, is unknown. Additional to this, plasticization, which is often talked about in the literature, where the hydrogen bonding is switched from cellulose–cellulose interactions to cellulose–water interactions is commensurate with a dramatic increase in the free water.^134^ It has been shown that it is the bound water that indeed breaks the hydrogen bonding between cellulose,^135^ which begs the question as to whether it is just hydrogen bonding at play in switchable nanocellulose-based composites?

### 4.1 Water Responsive Cellulose-Based Nanocomposites

**Figure 12:**
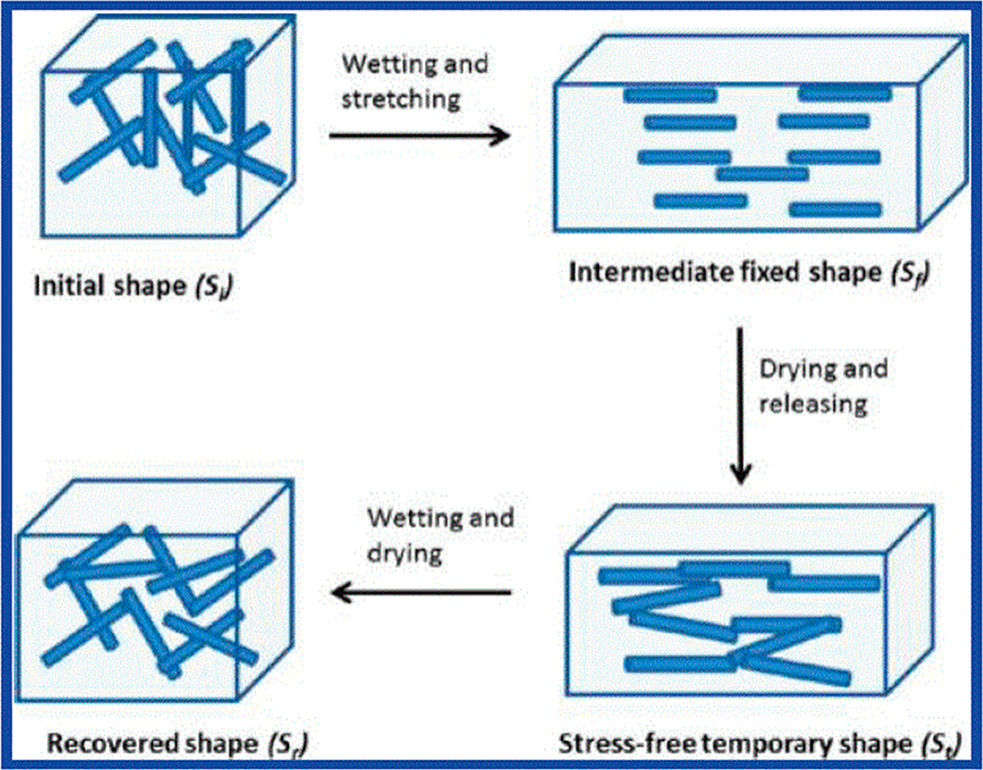
Schematic representation of the shape-memory effect within a polyurethane (PU)/cellulose nanocrystal (CNC) showing a cycle of wet-stretching, drying, and releasing and finally wetting and drying. Reproduced with permission from ref (37). Copyright 2011 American Chemical Society.

One example of a highly impactful study on stimuli-responsive nanocomposites based on cellulose was by the group of Christoph Weder, and others, where they showed that the addition of water as a “chemical regulator” to an ethylene oxide–epichlorohydrin/CNC nanocomposite resulted in a significant reduction in the stiffness of the material (from 800 to 20 MPa on immersion in deionized water).^136^ Furthermore, this effect was demonstrated to be wholly due to the disruption of the internetwork bonding (assumed hydrogen bonding) between CNCs inside the composite, and not matrix plasticization.^136^ This was demonstrated by swelling the materials in 2-propanol (IPA), which is known to have a similar effect to water; no decrease in the mechanical properties was however observed. This unique interplay between water and cellulose further enabled switchable interfaces in other nanocomposites, including with polyurethane (PU), demonstrating shape-memory deformation.^6^ The shape-memory effect relied on the ability to disengage the interactions between a network of CNCs within the PU matrix, enabling an orientation of the rod-like particles Figure 12.^37^ Upon drying, the CNCs remained in this oriented state, only relaxing again upon further addition of water whereupon the network reforms its shape and connectivity.^37^ Similar results were also obtained by another group, again showing shape-memory effects.^137^

### 4.2 Percolation Models of Interaction and Water Responsiveness

**Figure 13:**
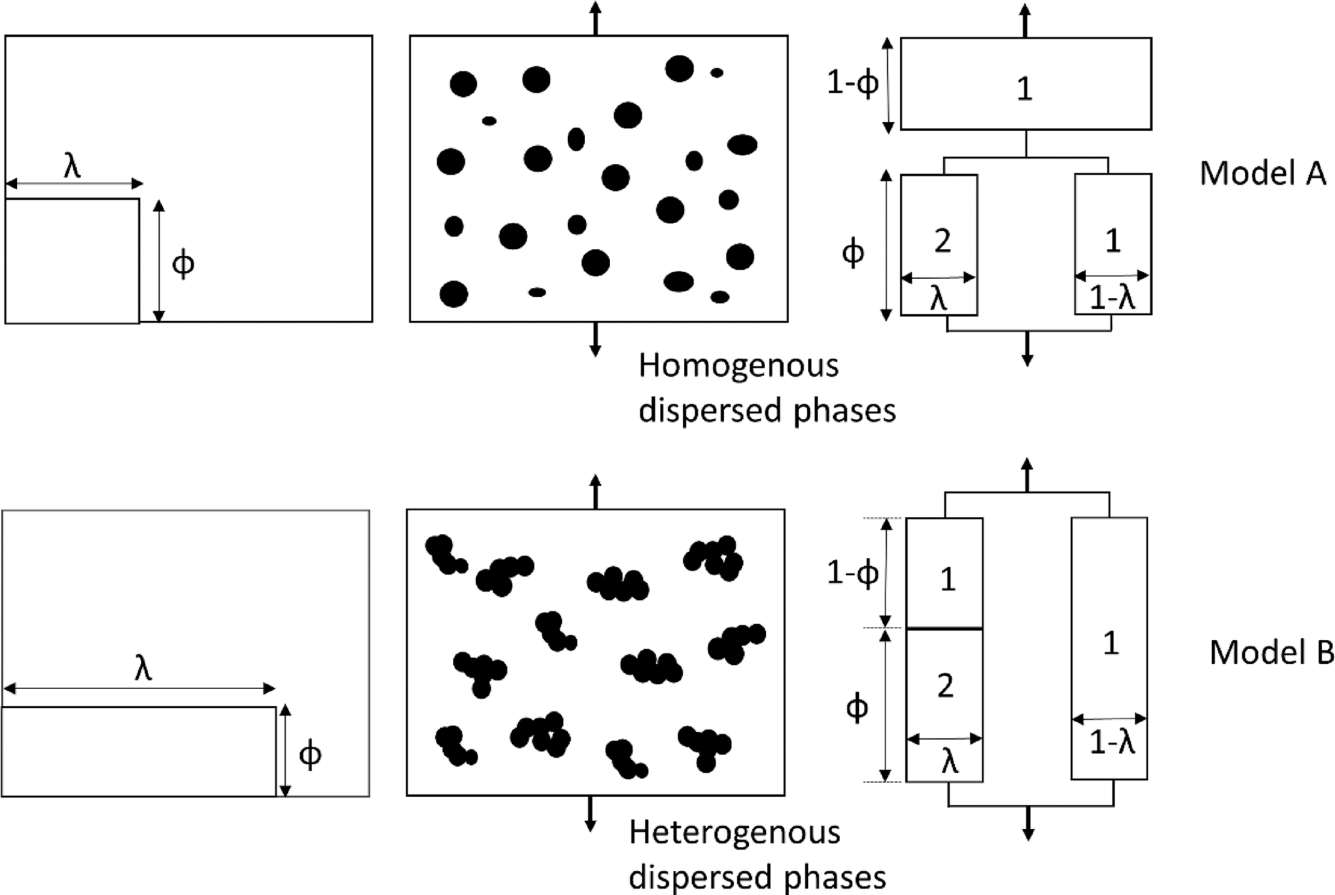
(from left column to right) Equivalent models (top and bottom; left) for homogeneous and heterogeneous dispersions (top and bottom; middle) of crystalline domains with phenomenological and equivalent series-parallel models of deformation. λ and Φ. Modified from ref (125).

**Figure 14:**
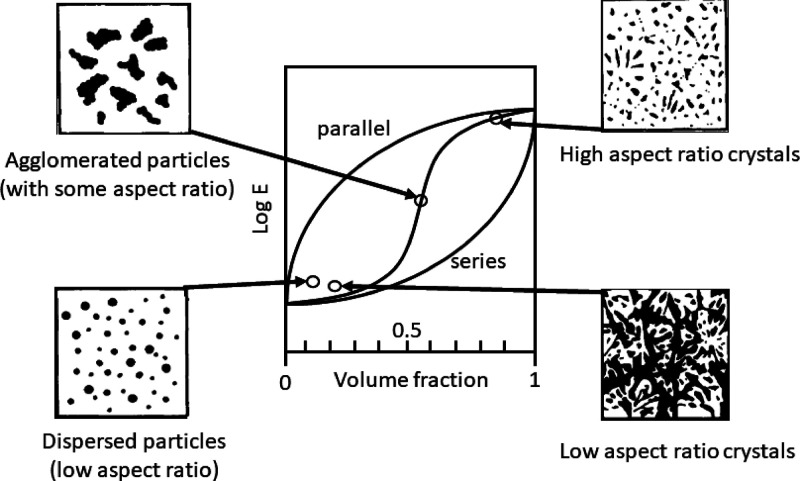
Schematic of the parallel (upper bound) and series (lower bound) models of reinforcement indicating representations of the different morphologies that give rise to mechanical behavior bound by these two limits. The graph shows modulus (*E*; on a log scale) as a function of volume fraction. Modified from ref (142).

More recently, some work by Bortner’s group^138^ has demonstrated that diffusion into a polymer matrix–CNC composite is increased with the addition of CNCs into a PU/CNC composite and that the dry and wet state mechanical properties can be modeled using percolation and Harpin–Kardos models, respectively. The percolation model is governed by the equation^136,139,140^
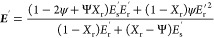
1with
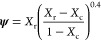
2where *E*′ is the storage modulus of the composite, *E*_s_′ and *E*_r_′ are the experimentally determined moduli of the matrix and the reinforcing phase, respectively, *X*_r_ is the volume fraction of the reinforcing phase, *X*_c_ is the critical percolation volume fraction, and ψ is the volume fraction of the reinforcing phase that take part in load transfer. It is worth going back to the original papers on these models, specifically the Takayanagi model,^139^ and the further developments by Ouali et al.^140^ for multiphase polymers, although their equation is largely based on the former. The models are phenomenological and based on viscoelastic constructions and variations of series-parallel models. They really comprise three phases, not the two (reinforcement and matrix) that might be assumed on first sight, with a series parallel arrangement as depicted in Figure 13(139,141) but representing a *dispersed* stiff phase in a more compliant one. In fact, the later model by Halpin and Kardos^142^ were critical of the use of a parallel phase (effectively the third phase in Figure 13), probably because it has no physical basis for its inclusion. Two models are proposed by Takayanagi et al.,^139^ both of which equivalently describe homogeneously dispersed and heterogeneous systems of phases. Halpin and Kardos^142^ point out that all forms of reinforcement (particle, platelet, needle) are bound by the upper (parallel, Reuss model) and lower (series, Voigt model), with S-shape laws of reinforcement for all composite structures placed in between these bounds (Figure 14). The upper bound curve is often depicted and described by a linear line and the equation for the rule of mixtures, whereas the lower bound is a curve. The mechanical performance of any polymer blend, composite, or fiber is bound by these two limits, and where it is placed within this depends on the morphology of the sample. Highly affine, high aspect ratio crystals in a softer matrix yield high stiffness. Low aspect ratio particles on the other hand offer little in the way of reinforcement.

One of the major issues with the use of these models is that being phenomenological, they have limited physical meaning beyond what has been already described. Their use has been proposed to describe the water interactions between CNCs within a network, but little has been done to fully interrogate the models and adapt them for this purpose. It is assumed that the models developed by Takayanagi et al. are best used for networks of CNCs, whereas Halpin–Kardos for disengaged networks when wet. However, given the 3-phase nature of the models (see Figure 13), it ought to be possible to adapt the Takayanagi model to account for wet and dry states of the network. In addition to this, the interface between the reinforcing phase (CNCs) and the matrix is assumed to also play a role in the compliance of a wet composite, something which is not really discussed in many of the published works on adaptive cellulose nanomaterial based composites. To address this it is perhaps better to return to the originally described models proposed by in a review of polymer blends by Dickie.^143^ They derived a formula for the shear modulus of a polymer blend, which we have adapted here to describe the elastic modulus (*E*) of a composite, as
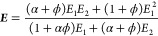
3where α is a connectivity parameter between the phases in the composite, ϕ is the volume fraction of the reinforcing phase, and *E*_1_ and *E*_2_ are the moduli of the matrix and the reinforcing phase, respectively.

**Figure 15:**
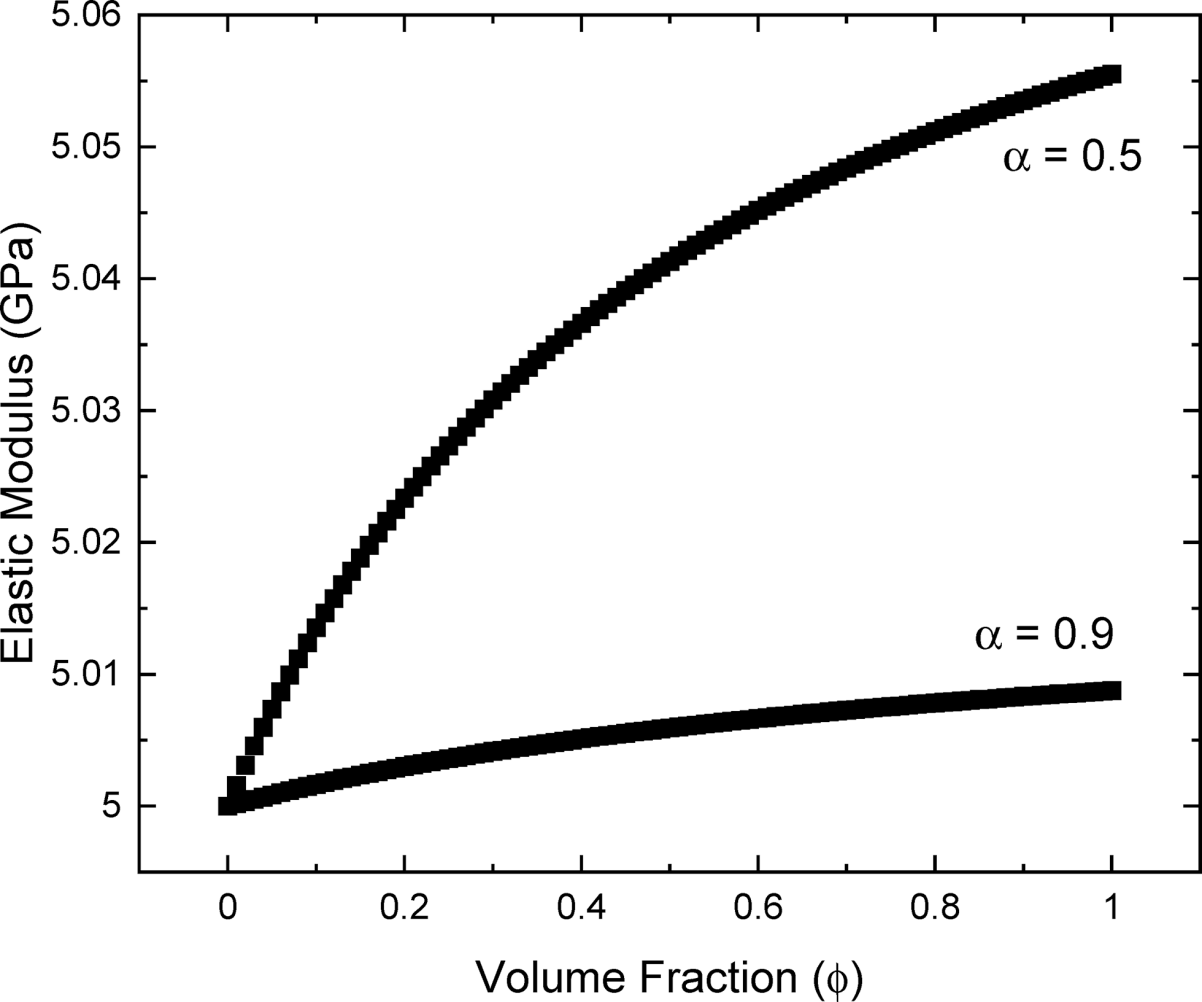
Example curves of the elastic modulus (*E*) as a function of volume fraction (ϕ) according to eq 3 for values of *E*_1_ = 5 GPa (typical for an epoxy resin) and *E*_2_ = 145 GPa (typical for a tunicate cellulose nanocrystal).

Using this equation to plot a range of curves for different values of α (Figure 15), it is clear that this function could easily account for the changes in the wet and dry states by simply having a different value of α. What is more, this would have physical interpretation in that the coupling parameter would include some idea of the interaction between the water and the CNC network, and additionally between individualized CNCs and a matrix phase, something which is not accounted for in the percolation model.

It is clear then that much simpler explanations for the rise in the modulus, both on account of intact network stiffness and interfaces with the resin, can be accounted for in this approach. Additionally, it is questionable that the initial “flat” region of the percolation model is indeed represented in most data for cellulose nanocrystal based composites, with it more likely being a continuous increase from the modulus of the matrix to the reinforced state. Judicious use of the term α can account for wet and dry states. It is also possible to address the interaction between the matrix and the stiff reinforcing phase through the use of a contiguity parameter, ξ, as has been suggested before by Ganster et al.^144^ and originally proposed by McCullough et al.^145^ This approach often gives reinforcement curves that sit between the upper and lower bounds (cf. Figure 14) and have previously been adapted and applied to natural fibers,^146^ where it is proposed that there are crystalline domains contained within an amorphous matrix but without periodicity.^145^ The contiguity parameter itself is related to the aspect ratio of the reinforcing phase, and values of around 5 have been used for affine reinforcement from cellulosics.^146^

### 4.3 Hygromorphic Responsive Cellulose-Based Composite Hydrogels

Another significant area of research within the field of water responsive cellulose based composites is the concept of a 4D shape-changing hydrogel system, so-called hygromorphic systems. These systems gain inspiration from natural structures, such as the leaves on plants, and wheat awns which change shape in response to water due to differential swelling based on alterations of the orientation of the structures.^147^

**Figure 16:**
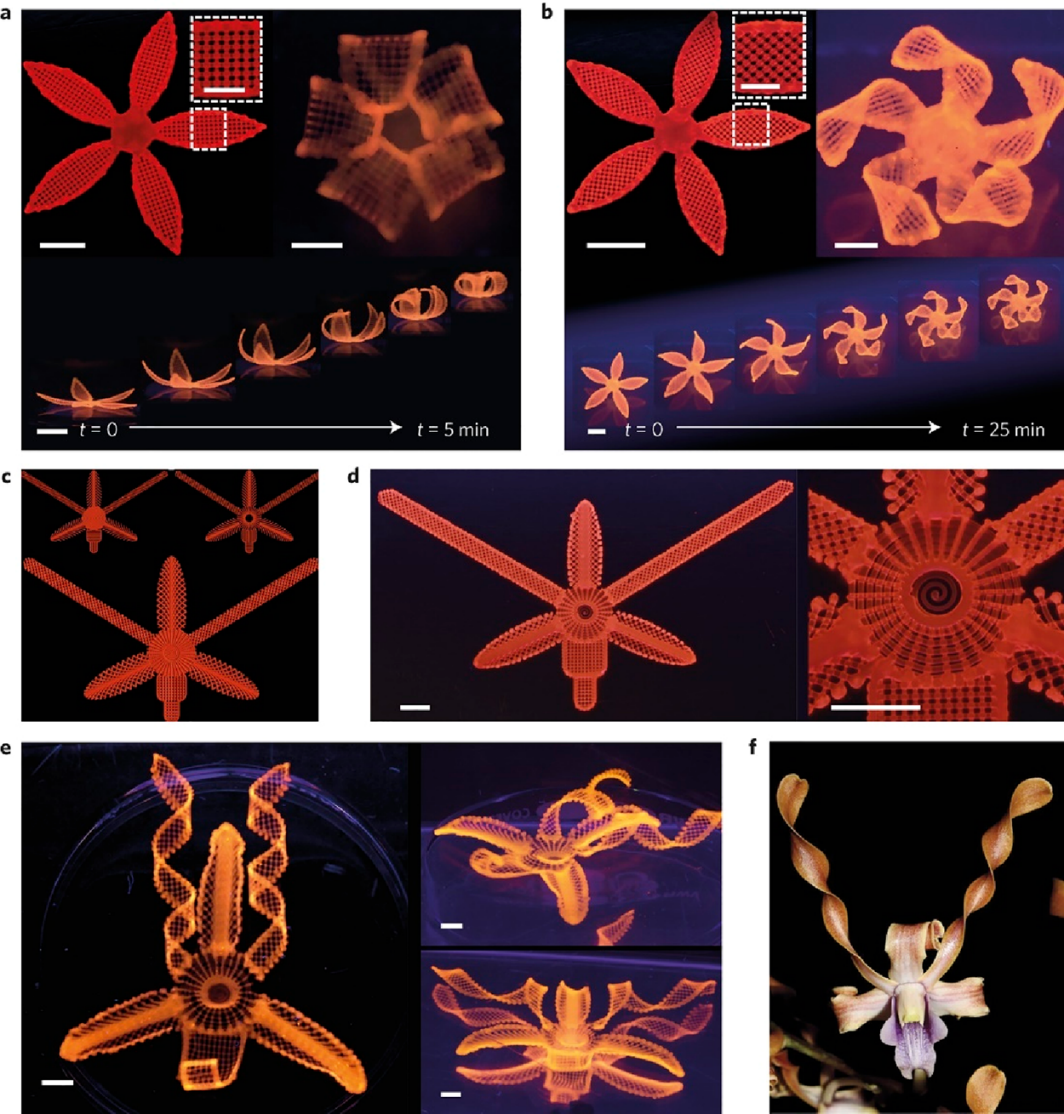
Examples of hygromorphic plant like structures made using polyacrylamide/cellulose nanofibril composite gel materials; simple flower structures printed with (a) 90°/0° and −45°/45° ply structures, with the angles representing the orientation of the nanofibrils with respect to the long axis of each petal; the time axis shows the change in shape on immersion in water during the swelling process, (c) the print path, (d) the resultant swollen structure, and (e) a swollen structure based on the native orchid (*Dendrobium helix*) as shown in (f). Reproduced with permission from ref (148). Copyright 2016 Nature.

Such a system was first conceived by Jennifer Lewis’ group.^148^ In this work, they printed a cellulose fibril/soft acrylamide composite gel system, incorporating directionality to the fibrils, but also other components (clay, photoinitiator, glucose oxidase, glucose) to assist with printing and curing. Shape changing biomorphic structures (Figure 16) were obtained, which responded to changes when submerged in water.^148^ These changes in shape were demonstrated for a range of different systems, even showing how complex flower morphologies can be mimicked.

Similar results have been subsequently obtained for a hydrogel comprising pulp fibers in a carboxymethyl cellulose matrix,^149^ although it could be argued that this system is less complex and as it primarily contains cellulose will inevitably represent a clearer interaction of the material with water. This whole subject of hydrogels that respond to external stimuli is an area of research in its own right, and the readers are referred to further reviews.^150^

Returning to the natural systems that display changes in shape upon actuation in water, a finite element modeling study by Zickler et al.^151^ showed that the probable mechanism for the swelling and shape changes in wheat awns is the formation of gaps between laminations, which act as valves, allowing moisture into the cell walls. This interplay of structure of laminations, and the molecular level of cellulose and its interactions with water demonstrate that there are most likely hierarchical features that lead to macroscopic behavior. The ability to mimic such structures, and thereby morphing characteristics, remains a wide-open topic of research.

## 5 Cellulose Used in Water Filtration

At the molecular level, several intrinsic properties, particularly the potential for surface modifications, e.g., with amines, phosphates, and carboxyl groups, also make nanocellulose particularly attractive for water treatment applications as such modifications improve its interactions with water and contaminants.^152^ With respect to cellulose derivatives used for water filtration, cellulose acetate and other derivatives are important materials. Indeed, there have even been some commercial regenerated cellulose-based products for extraction of viruses, such as Planova by Asahi Kasei,^153^ and a wide range of cellulose esters made by Eastman.^154^ A full description of these technologies is however beyond the scope of this article because we mostly deal with nanocellulose.

Nanocelluloses may also be used to improve the physical and chemical properties of membranes and adsorbents, i.e., mechanical strength, hydrophilicity, permeability, selectivity, and biofouling resistance^155^ and nanofibrils, due to their tendency for entanglement can stabilize catalysts,^156^ and improve the structural integrity of aerogels.^157^ As with other nanomaterials, nanocelluloses also present large specific surface areas and a greater number of active sites, making them superior adsorbents for contaminants. This section examines how these qualities of nanocellulose have been exploited in water treatment strategies ranging from adsorption, absorption, membrane filtration, and catalytic degradation. Using tools including the hard soft acid base theory, we demonstrate how contaminant capture can be optimized by appropriate surface functionalization. Finally, we also assess the potential of less reviewed approaches of nanocellulose used in water treatment e.g., solar evaporation, reducing end modifications.

### 5.1 Nanocelluloses As Adsorbent Materials

Adsorption is a surface phenomenon in which the contaminants (adsorbates) from the surrounding media interact with the adsorbent surface. The interactions are governed by π–π interactions, forces that produce physical bonds between contaminants and the adsorbent surface, e.g., van der Waals forces, hydrogen bonding, as well as chemical bonds, e.g., ion exchange and complexation.

The application of nanocellulose as the active adsorbent is based on the presence of appropriate surfaces which functionalities can be imparted, through approaches broadly classified as (i) direct modification of the surface, e.g., through acid hydrolysis, TEMPO oxidation, periodate oxidation, or (ii) grafting of polymers with the desired functionalities.^158−162^ Surface modification by acid hydrolysis often results in the modification of C-6 OH groups, as these are most accessible, although modification of C-3 and C-4 OH moieties have also been reported, e.g., following phosphoric acid hydrolysis.^163^ Finally, a less explored approach to surface modification involves reducing end modification^158,160^ which offers the possibility of imparting dual functionalities on a single anhydroglucose unit through the C6 OH group, thereby increasing adsorption efficiency.

Surface charges play a significant role in the interactions between adsorbates and adsorbent surfaces. As such, it is important, in the design of adsorbents, to consider the nature of the target contaminant. Positively charged contaminants, e.g., metal ions and cationic dyes, are efficiently captured using adsorbents with negatively charged surface groups, e.g., carboxylates (−COO^–^), phosphates (PO_4_^3–^), and thiol (−SH) groups. The capture of anionic contaminants, on the other hand, is promoted by positively charged surface groups which can be imparted through amination and quaternisation reactions.

Pearson’s hard soft acid base (HSAB) concept also offers meaningful guidance in selecting surface modifications to impart to nanocellulose surfaces. Soft acids form more stable covalent bonds with soft bases, hence improving the removal of Hg(II), Ag(I), and Au(I) ions by thiolate and thiocyanate containing adsorbents. While hard acids such as Cr(III), Cr(VI), Mg(II), and Fe(III) ions are better adsorbed by hydroxides, carboxylates, and ammonia groups (hard bases). Borderline elements, such as Fe(II), Co(II), and Pb(II), are best removed using adsorbents with pyridine, aniline, nitrate, and sulfate moieties.

#### 5.1.1 Carboxylation

Carboxylation is one of the most studied surface modifications of nanocellulose materials. Carboxylated CNCs and CNFs can be prepared using a variety of oxidants including 2,2,6,6-tetramethylpiperidine-1-oxygen (TEMPO),^164^ ammonium persulfate (APS), hydrogen peroxide,^165^ and periodate–chlorite combination,^166^ anhydrides of succinic, phthalic, and maleic acids,^167^ as well as organic acids, e.g., oxalic acid^168,169^ and citric acid.^170^ Organic acid derived materials seem to have higher aspect ratios compared to CNFs generated using sulfuric acid,^170^ i.e., aspect ratios of 144 versus 83, which is beneficial for aerogel formation by physical bonding, precluding the use of chemical cross-linkers. Dye (methylene blue, MB) and metal (Cu^2+^) removal by the physically cross-linked aerogels was 132.98 and 45.05 mg g^–1^, respectively. Similar removal efficiencies: 110 and 51.1 mg g^–1^ for MB and Cu^2+^, respectively, were reported for CNCs with a carboxylic acid content of 2.2 mmol g ^–1^, that had been prepared by hydrogen peroxide oxidation.^165^ Carboxylation by this approach is driven by H^+^ ions and OH• generated from Fe^2+^ catalyzed degradation of H_2_O_2_. H^+^ ions protonate the β(1→4) glycosidic bonds resulting in its disintegration, while the free radicals attack the OH groups to produce carboxylic acid groups. Adsorption of dyes is then driven by hydrophobic ring–ring interactions with the cellulose molecules, as well as electrostatic interactions between cationic centers on the MB ring and the carboxylate groups on CNCs. This may explain the higher adsorption of MB relative to copper, as only a single mechanism is involved in uptake of the latter.

Sehaqui et al.^171^ examined the use of cyclic anhydrides as oxidizing agents in the preparation of CNFs from wheat fibers. CNF prepared with succinic anhydride (S-CNF) generated the highest concentration of COO– groups (3.8 mmol g^–1^), much higher than those of CNFs prepared using maleic anhydride (0.9 mmol g^–1^) and phthalic anhydride (1 mmol g^–1^). When added to paper filters at a 5 wt % concentration, S-CNF led to an increase in Pb^2+^ removal from 50 to 96.5%.

The production of CNF by nitro-oxidation was popularized by Hsiao’s group at Stony Brook University.^172,173^ It is considered a greener approach because, besides precluding the traditional pretreatment steps of bleaching and delignification, waste products of the process can be converted to fertilizer, thereby reducing chemical and water consumption. The process involves reacting nitric acid and sodium nitrate in the presence of excess acid to generate the oxidizing agent: nitroxonium ions (NO^+^), which then attack the primary hydroxyl group of cellulose to generate carboxylate groups. Chen et al.^174^ used this approach to isolate CNFs from Moringa plants before applying them for Hg^2+^ adsorption. Nanofibers 10–12 nm in width and 250–300 nm in length, with 0.97 mmol g^–1^ carboxylate content, were obtained. The maximum Hg^2+^ adsorption capacity for the CNFs was 257.07 mg g^–1^. Drying the CNFs reduced Hg^2+^ capture slightly, from 81.6 to 74.3%, suggesting that drying for economically viable transportation does not substantially reduce performance of the adsorbent. Higher capacities were, however, reported when adsorbents prepared by this approach were applied for removal of UO_2_^2+^ and Cd^2+^ions, i.e., 1470 and 2550 mg g^–1^, respectively.^172,173^

**Figure 17:**
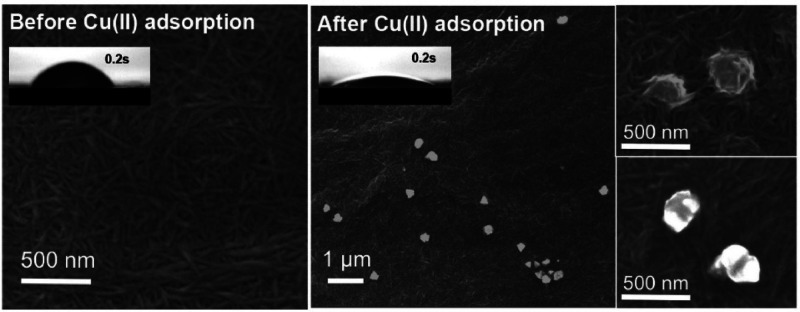
SEM images of TOCNF casting film and TOCNF filter cake before and after Cu(II) adsorption, showing the formation of nanosized clusters of Cu/CuO on CNFs. Reproduced with permission from ref (175). Copyright 2015 Elsevier.

An interesting finding regarding the effect of sorption on the water flux of membranes made from TEMPO-oxidized CNF was reported by Liu et al.^175^ Cu^2+^ ions adsorbed onto CNFs were reduced to Cu^0^ and CuO nanoparticles (Figure 17), resulting in increased flux. Multiple mechanisms were suggested for this effect. First, crystallization of the adsorbed ions resulted in fibers pulling together, increasing the mean fiber diameter, and creating voids that increased pore diameter, and subsequently, increasing membrane flux. The second mechanism was by increasing hydrophilicity. Filtration of a 200 ppm of Cu^2+^ solution decreased the contact angle from 46.3° to ∼18°, thereby improving water flow through the membrane. Considering the antibacterial properties of CuO NPs, this approach seems to present triple benefits for membranes. An ion that may come in as a contaminant could be trapped, increase membrane flux, and exert an antimicrobial effect.

Finally, carboxylated nanocellulose can be used for adsorption of organic contaminants including pharmaceutical drugs and pesticides. TEMPO–CNF were covalently attached to Jeffamine ED 600 (*O*,*O*′-bis(2-aminopropyl) polypropylene glycol-*block*-polyethylene glycol-*block*-polypropylene glycol) and used for the adsorption of acetaminophen, sulfamethoxazole, and *N*,*N*-diethyl-*meta*-toluamide (DEET).^176^ The binding of Jeffamine ED 600 to CNFs decreased the zeta potential of the composite. As such, at low pH where unbound carboxylate groups of CNF were protonated, the adsorbent was uncharged, and aggregated, leading to low adsorption. At higher pH, deprotonation and charged surfaces allowed for distribution of the adsorbent in solution. Similar findings were reported for the adsorption of salbutamol (a bronchodilator) by succinylated CNFs.^177^ As salbutamol existed largely as protonated or zwitterionic ions below pH 11, its adsorption was dependent on changes in the surface charge of CNFs. Only when the pH increased above 7 and they became deprotonated was adsorption of the positively charged salbutamol species possible. An important note for covalent bonding in adsorbent synthesis: while it ensures more stable cross-linking, it also uses up the reactive carboxylate groups required for adsorption. As such, a robust adsorbent with poor efficiency may be created.

#### 5.1.2 Thiolation

Various thiolating agents have been explored for functionalization of nanocellulose, including (3-mercaptopropyl)-trimethoxysilane (MPTMS),^178^ thiourea,^179^ thioglycolic acid,^180^ 3-mercaptopropionic acid,^181^ and l-cysteine.^182^

**Figure 18:**
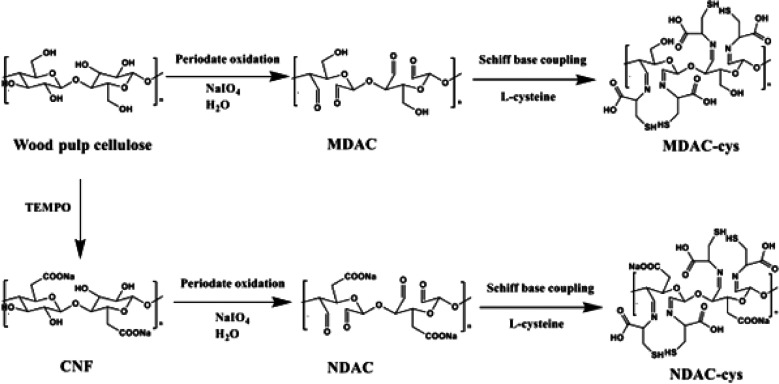
Reactions for periodate oxidation and l-cysteine modification of microscale and nanoscale cellulose. Reproduced from ref (183) (CC-BY image).

Based on the HSAB theory, thiol groups are soft bases which makes them appropriate for capture of soft cations such as Hg^2+^ and Pb^2+^. Geng et al.^178^ showed that the adsorption of Hg^2+^ was twice as fast with aerogels made from MPTMS-functionalized CNFs than with TEMPO oxidized CNFs, i.e., 5 h versus 10 h. Importantly, CNFs without MPTMS had a removal efficiency of 23%, demonstrating that carboxylate groups are also involved in Hg^2+^ capture, a finding confirmed also by XPS analysis. The calculated maximum adsorption capacity of the thiol-modified TEMPO CNF of 729.9 mg g^–1^ was close to the experimental value of 718.5 mg g^–1^. Li et al.^182^ reported an even higher adsorption efficiency (923 mg g^–1^) using l-cysteine-modified CNCs. In their approach, CNCs generated from sulfuric acid hydrolysis were oxidized using sodium periodate to generate dialdehyde CNCs. Aldehyde groups were then reduced by using sodium cyanoborohydride (NaBH_3_CN) before reaction with l-cysteine (Figure 18). Complexation by cysteine NH and SH groups in the adsorbent likely improved the removal efficiency. However, Hg removal reduced drastically (55%) after the fourth cycle, likely due to strong thiol–Hg^2+^ bonds.

Some findings with relevance to the design of adsorbents for water treatment were reported by Chen et al.^183^ This study compared l-cysteine functionalized micro- and nanofibers for As(III) removal. Both materials were functionalized with l-cysteine by Schiff base coupling after the periodate oxidation, resulting in attachment of thiol moieties at C-2 and C-3, but this difference was lower than would be expected considering that TEMPO CNFs (∼5 nm in diameter) are 6000 times smaller in diameter than typical microfibers (∼30 μm in diameter). Thus, theoretically, the surface area for functionalization per gram of adsorbent is far greater for CNFs. However, cysteine content was 648 mg g^–1^ in the nanofibers and 497 mg g^–1^ for the microfibers.^183^ Further, As(III) removal by the two materials also did not vary much: 344.82 versus 357.14 mg g^–1^ for the micro- and nanoscale materials, respectively. Neither did the temperature at which thermal degradation began: 101 °C for CNF and 115 °C for microfibers. Together, these data suggest that nanofibers may not always present considerably higher adsorption capacities, and it may be worthwhile considering microscale materials whose preparation involves fewer steps and reagents. Periodate oxidation may lead to partial defibrillation in microfibers resulting in a material with large inner surface areas for functionalization.

Thiol-modified nanocellulose has also been investigated for selective flocculation of chalcopyrite and pyrite minerals.^184^ Silylated CNFs were modified using MPTMS.^185^ The materials had a turbidity removal efficiency of 90–99% at a concentration of 4000–8000 ppm and showed high selectivity for chalcopyrite and pyrite as removal of quartz particulates was only 30%. Silylated CNFs could therefore potentially be useful in the treatment of surface water around mine dumps that are often contaminated by fine particulates deposited by wind or by rainfall driven attrition of waste heaps.

#### 5.1.3 Cationization

Modifying nanocellulose surfaces with cationic groups allows for the adsorption of a broad range of negatively charged contaminants including anionic dyes,^186^ pharmaceutical drugs,^187^ pesticides,^188^ chromates,^189^ and other anions (phosphates, nitrates, and sulfates).^190^ Different methods and a variety of cationization agents have been reported, with varying degrees of success. It is worth noting here that while addition of amino functionalities, e.g., using polyethylenimine (PEI) also leads to cationic surfaces, the surface charge is pH-dependent, and so amine functionalized materials are only positively charged at low pH.^191^ Tertiary amine groups of PEI (2 ≤ p*K*_a_ ≥ 3) are protonated at a pH of 2.^192^ Below this pH, therefore, CNF surfaces are positively charged and would repel rather than adsorb cations. In contrast, quaternary ammonium compounds (QACs)^193^ are positively charged over a wide pH range, e.g., 3–8, and are, therefore, more attractive for cationic functionalization. QACs reported in the literature for functionalization of nanocellulose include Girard’s reagent T ((2-hydrazinyl-2- oxoethyl)-trimethylazanium chloride),^194,195^ epoxy-propyltrimethylammonium chloride (EPTMAC),^196,197^ glycidyltrimethylammonium chloride,^186,189,190,198^ imidazolium,^199^ aminoguanidine,^200,201^ pyridinium,^202^ and a deep eutectic solvents made from aminoguanidine hydrochloride and glycerol^203^ or boric acid and glycidyl trimethylammonium chloride.^204^

In water treatment applications, cationic nanocellulose is attractive because it offers ease of adsorbent recovery after adsorption. In a recent study, cationized cellulose was recovered after the adsorption of Cr(VI) ions by decanting or light centrifugation.^205^ This was possible because the adsorbents lost their surface charges once saturated with the contaminant, leading them to settle out of solution. Similarly, the formation of flocs with lateral dimensions of several millimeters when cationic cellulose was used for the flocculation of kaolin has also been reported.^195^ As the flocculation performance of cationic cellulose has already been shown to be better than that of commercial polyacrylamides,^204^ it is plausible that if produced via economically viable methods, it could replace polymeric flocculants in conventional water treatment.

One of the main challenges faced in aqueous cationisation approaches, however, is how to control the amount of water to achieve a high degree of substitution. Of course, one of the other issues of adding cationic groups is that you often immediately get adsorption of anionic polyelectrolytes, which can effectively neutralize the surface. The process is usually carried out in the presence of water and NaOH, with the former acting as a nucleophile to activate cellulose OH groups toward etherification. However, water promotes the formation of side reactions, reducing the efficiency of etherification and, subsequently, the degree of substitution (DS). Odabas et al. attempted to address this challenge by substituting some of the water with 2-propanol or THF and found that this increased the DS. Replacing up to 90% of the water with THF increased the DS from 0.05 to 0.35.^198^ A second approach reported by Zaman et al.^198^ involved mixing powdered CNCs with NaOH before the addition of water or a water/dimethyl sulfoxide (DMSO) mixture. Glycidyltrimethylammonium chloride (GTMAC) was then added dropwise to the mixture before heating at 60 °C for 4 h. They found that CNCs made using the DMSO process had much higher surface charge density (2.05 mmol g^–1^) than CNCs cationized in water (0.35 mmol g^–1^). The maximum fraction of water in the water/DMSO mixture for optimal cationization was determined to be 36 wt %. Together, these studies show substitution of a fraction of the water with organic solvents can improve cationization efficiency.

A third and more environmentally friendly solution is presented by the use of deep eutectic solvents (DES). Using an aminoguanidine hydrochloride and a glycerol DES system, CNFs and CNCs with a charge density of 2.48 mmol g ^–1^ were synthesized after treatment at 80 °C for 10 min.^203^ This surface charge was more than two times greater than when the same cationizing agent was used in a heterogeneous system, i.e., 1.07–1.70 mmol g^–1^.^201^ The DES could also be reused five times before replenishing of aminoguanidine was required. Deep eutectic solvents, therefore, provide a more efficient and sustainable approach to cationisation.

#### 5.1.4 Phosphorylation

Although relatively less explored in comparison to other functionalization approaches, addition of phosphate functionalities has significant benefits for water treatment, ranging from uptake of hard metal ions, e.g., lanthanide, actinide, and transition metal ions.^206−212^ Phosphorylation is also known to improve thermal resistance^213^ and may therefore be used to improve the thermal stability of membranes intended for high temperature applications.

Phosphorylation of native nanocellulose is an esterification reaction that involves mostly the C-6 hydroxyl group due to its greater nucleophilicity. Nevertheless, Lemke et al.,^163^ showed by using NMR studies that hydroxyl groups at C-2 and C-6 may also be phosphorylated. Various phosphorylating agents have been reported, including phosphorous acid (H_3_PO_3_),^214^ orthophosphoric acid (H_3_PO_4_),^210,215,216^ phosphorus pentoxide (P_2_O_5_),^217^ phosphorus oxychloride (POCl_3_),^218^ and sodium or ammonium phosphates, e.g., NaH_2_PO_4_,^219^ NH_4_H_2_PO_4_,^220^ (NH_4_)_2_HPO_4_,^221−223^ or other agents, including cyclotriphosphate (Na_3_P_3_O_9_)^209^ and hexachlorocyclotriphosphazene (P_3_N_3_Cl_6_).^218^ Shi et al.^224^ also reported functionalization with phosphate esters prepared by the reaction of 1-octanol or 1-octadecanol with P_2_O_5_.

Phosphorylation reactions may be classified as homogeneous or heterogeneous. The former, excluding water and phosphorylating agents, e.g., metaphosphoric acid are simply dissolved in molten urea.^215,225^ Heterogenous reactions involve solvents such as water,^215,225^ pyridine, hexanol, dimethylformamide (DMF), and tetrahydrofuran.^216,226^ To improve the degree of substitution, some workers have included a swelling pretreatment step using NaOH,^227,228^ but urea,^210^ DMF, and hexanol have also been reported, although the latter results in degraded end products.^216^

Urea plays a key role in the nanocellulose phosphorylation reaction; some studies have reported little or no phosphorylation when urea was left out of reactions.^210,217,229^ It seems to play multiple roles, including as a swelling agent, a solvent, as well as protecting fibers against excessive degradation.^230^ Granja et al.,^216^ for example, found that while high phosphorylation efficiencies were possible in the absence of urea, the yield was low due to dissolution of the cellulose fibers. Finally, recent work has suggested that urea does not simply act to create basic conditions in phosphorylation reactions. Blilid et al.^218^ used K_2_CO_3_ in the phosphorylation of nanocellulose with phosphorus oxychloride (POCl_3_) and found that the presence of this base hardly improved the success of the phosphorylation, i.e., 0.29–0.31%. Urea, therefore, seems to exert additional advantages besides a basic environment. Akin to NaOH, it likely breaks down van der Waals and hydrogen bonds in the fibrils, thus facilitating exposure to phosphorylating agents. Together, these data suggest that urea plays a key role in both homogeneous and heterogeneous phosphorylation reactions.

Not all urea containing molecules facilitate phosphorylation. Sterically hindered derivatives, e.g., 2-imidazolidone and tetramethyl urea, has resulted in far lower phosphorylation (170 ± 57 and 660 ± 4 mmol g^–1^, respectively) because of the lower number of N–H groups available for hydrogen bonding with cellulose. In contrast, using urea resulted in phosphate concentrations as high as 3300 ± 160 mmol g^–1^ and a degree of substitution of 26 ± 1%.^217^

Heterogeneous reactions tend to have lower phosphorylation efficiencies than homogeneous reactions, which has been linked to the presence of water.^215^ This is because water is a reaction byproduct, and its presence or accumulation favors the backward rather than forward reaction. In heterogeneous reactions, therefore, removal of water, e.g., by heating in a vacuum oven may lead to higher degrees of substitution (DS). Higher temperature also leads to higher DS. Increasing the reaction temperature from 85 to 105 °C increased the DS from 0.6 to 2.5, and the reduction of reaction time from 6 to 2 h.^59^

Phosphorylated cellulose has high adsorption capacities for a range of ions, including UO_2_^2+^,^211^ Ho^3+^, Sm^3+^, La^3+^, Cu^2+^, Cd^2+^, Co^2+^, Zn^2+^, Cd^2+^, Ni^2+^,^206^ Fe^3+^, Fe^2+^, Bi^3+^, and Ag^+^.^209^ Despite the high adsorption capacities of the adsorbent prepared by Inoue et al., e.g., up to 5727 mg g^–1^ for Fe^2+^ ions, the long reaction times required for its preparation (14 days at 50 °C) make the approach rather impractical. Nevertheless, the ion affinity was found to be in the order: Bi^3+^ > Fe^3+^ > Fe^2+^ > Ag^+^. What seemed to be contradicting findings with respect to Ag^+^ and Fe^3+^ ions were later reported by Liu et al.,^209^ i.e., that selectivity was higher for Ag^+^ than Fe^3+^ in both single ions and mixed ion solutions and that Ag^+^ adsorption attained equilibrium in half the time it took Fe^3+^ ions, i.e., 6 h versus 12 h, respectively. At face value, it seems paradoxical that a soft acid (Ag^+^) was adsorbed to a hard base (phosphate) and faster than a hard acid (Fe^3+^). The answer, however, may lie in the fact that at the 62.5 mg L^–1^ concentration used for both ions, far more Ag^+^ than Fe^3+^ ions were present in solution. In fact, based on their molar masses, the concentration of ferric ions was about half that of silver ions. Silver ions, therefore, benefitted from a greater drive to adsorption sites than the ferric ions, leading to a greater adsorption for Ag^+^ in both single ion and mixed ion solutions. Where the authors used molar concentrations and concentrations were comparable, i.e., 55 mol Fe^3+^ and 63 mol Cu^2+^ ions, adsorption followed the HSAB theory. Adsorption of cupric ions by phosphate functionalized CNFs was somewhat subdued (19.6 mg g^–1^) and ferric ions were preferentially adsorbed. Uptake of uranyl ion, a hard metal, by phosphate-modified CNFs was also high as 1550 mg g^–1^.

Finally, despite being good chelators, phosphate groups are relatively unstable to hydrolysis.^231^ As such, the more stable phosphonates, despite being less efficient in adsorption, may be worth considering. Phosphonated nanocellulose can be synthesized by reacting dialdehyde cellulose with sodium alendronate,^232^ and an adsorption capacity of 1.98 mmol g^–1^ for vanadium ions has been reported for this approach.^208^

#### 5.1.5 Polymer Grafting

Polymer grafting allows for the covalent attachment of polymer chains to CNCs and CNF. Grafting approaches are broadly classified as being either grafting “to”, where a preformed polymer is attached to the nanocellulose surface, or grafting “from”, where polymerization is initiated at the surface of the CNC or CNF. Each approach has advantages and disadvantages. While grafting to allows for aqueous synthesis and thorough characterization of the polymer before grafting, steric hindrance from long or entangled polymer chains often limits the extent of polymer attachment in this approach, resulting in lower grafting density. Grafting from approaches, on the other hand, result in higher grafting density, but involves multiple synthetic steps, and poses challenges with characterization of the attached polymers, e.g., length. The approach also involves side reactions that produce free/ungrafted homopolymers that are difficult to separate from modified materials, leading to impure products.

Grafting to can be by (i) carbodiimide coupling, where carboxylate groups on nanocellulose react with amine groups of polymers being grafted, (ii) epoxy ring opening, where deprotonated surface hydroxyl groups react with the epoxy ring of the polymer, or (iii) isocyanate mediated, which is used to couple CNCs with hydrophobic polymers, e.g., caprolactone homopolymers,^233^ copolymers,^234^ and polyurethane.^235^ Grafting from approaches fall under three broad categories: (i) ring-opening polymerization, which is often used for grafting hydrophobic polymers, (ii) free radical polymerization, whose convenience lies in the fact that it can be performed in water and does not require prior attachment of initiators, and (iii) controlled radical polymerization (CRP), which is further divided into atom transfer radical polymerization (ATRP), reversible addition–fragmentation chain-transfer polymerization (RAFT), and nitroxide-mediated polymerization (NMP).^159^ Despite requiring prior attachment of an initiator and tedious postattachment purification steps to remove the catalyst, CRP techniques, particularly ATRP, are attractive because they enable control of the polymer length and grafting density. We present selected instances where grafting to and grafting from approaches for modification of nanocellulose have been used or present opportunities for water treatment applications.

**Figure 19:**
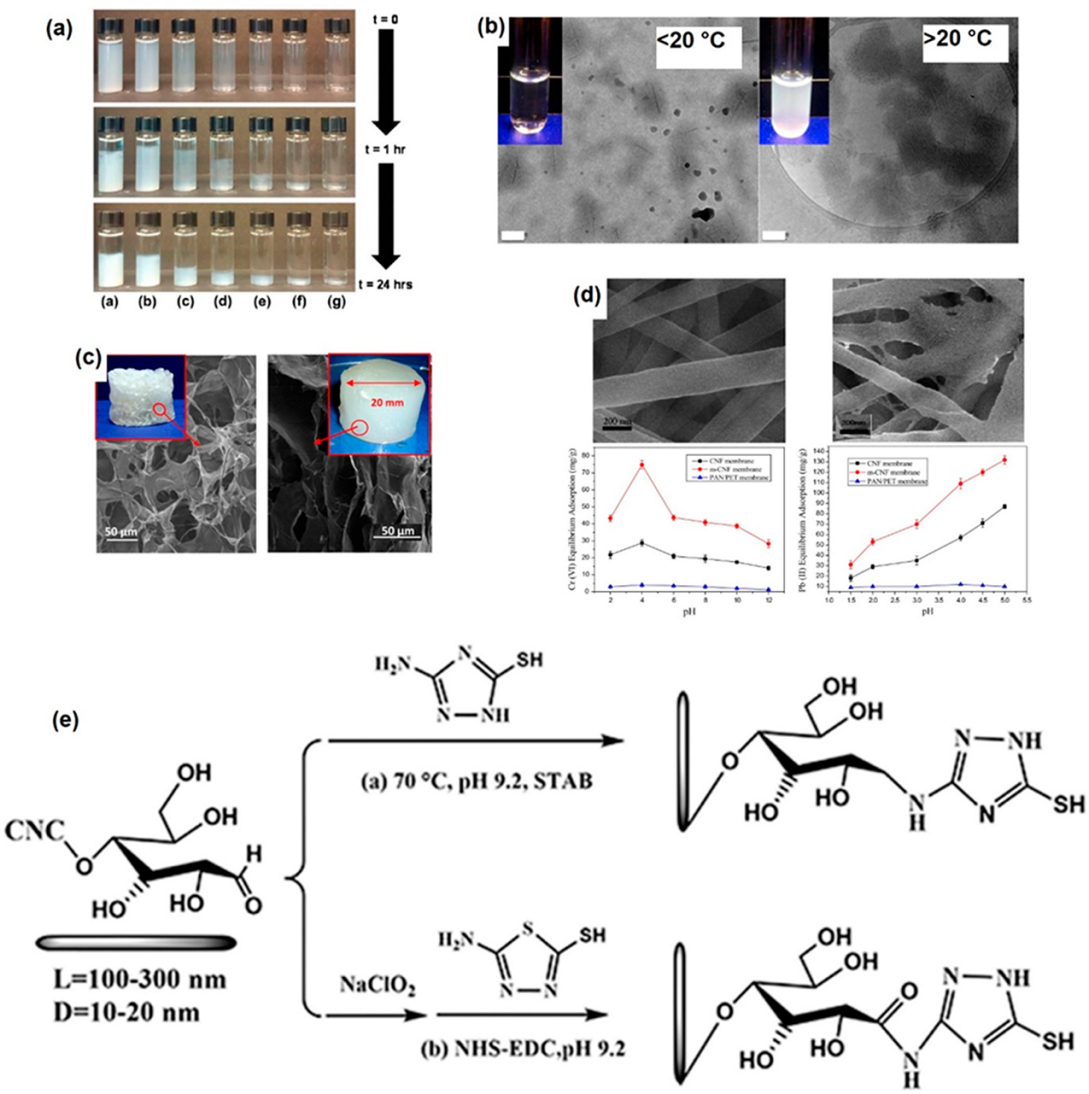
Flocculation behavior of poly(4-vinylpyridine)-modified CNCs at pH 10 immediately after pH adjustment, after 1 h and 24 h. Concentrations are as follows: (a) 0.250, (b) 0.150, (c) 0.050, (d) 0.025, (e) 0.013, (f) 0.006, and (g) 0.004 wt %. Reproduced with permission from ref (236). Copyright 2012 American Chemical Society. (b) Cryo-TEM images (insets) of temperature response behavior (below and above 20 °C) of CNCs modified with elastin-like polypeptides. Reproduced with permission from ref (237). Copyright 2018 American Chemical Society. (c) Scanning electron micrographs (inset) of nanocellulose aerogel before and after grafting of poly(methacrylic acid-*co*-maleic acid). Reproduced with permission from ref (238). Copyright 2015 Elsevier Ltd. (d) SEM images of the top views of an electrospun PAN nanofiber scaffold (a) and the scaffold with thiol-modified CNF (0.03 wt %). The effect of pH on adsorption of Cr(VI) and Pb(II) by unmodified electrospun polyacrylonitrile (PAN) membrane, unmodified CNF membrane, and PAN membrane with thiol-functionalized CNF. Reproduced with permission from ref (239). Copyright 2015 Elsevier Ltd. (e) Chemical routes for the grafting of triazole molecule on reducing end of CNCs to generate thiol-modified CNCs. Reproduced with permission from ref (240). Copyright 2018 American Chemical Society.

Kan et al.^236^ prepared pH-responsive flocculants by surface-initiated graft polymerization of 4-vinylpyridine, using ceric(IV) ammonium nitrate as an initiator. Because 4-vinylpyridine is hydrophilic in its protonated state (pH < 5) and hydrophobic in the deprotonated/uncharged state, grafted CNCs produced by this one-pot synthetic approach showed reversible flocculation and precipitation with changes in pH (Figure 19a). It is plausible that CNCs modified with this polymer could be useful in pH-controlled treatment of hydrocarbon-contaminated water. By raising the pH of the mixture above 5, the modified CNCs absorb the hydrocarbons and precipitate out of solution, leaving clean water as the supernatant to be decanted. Malho et al.^237^ reported dual functionalization of CNCs in an approach that could also have application for flocculant development. CNCs were coated with poly(acrylic acid) (PAA) and elastin-like polypeptides (ELPs). The resulting CNC–polymer complexes had both pH and temperature responsiveness, the former because of PAA and the latter due to ELPs. The carboxy groups of the PAA were protonated and positively charged at pH 3.0 but deprotonated and negatively charged at pH 8.4.

Similarly, the functionalized CNCs were agglomerated above 20 °C but formed a homogeneous dispersion below 20 °C (Figure 19b). Such dual response mechanisms allow for adsorption by flocculants via processes that could be controlled by either pH or temperature changes of the contaminated water.

Polymer modified nanocellulose has also been investigated for the treatment of metals and dye contaminated water. Maatar and Boufi^238^ synthesized a nanocellulose aerogel for the removal of Pb^2+^, Cd^2+^, Zn^2+^, and Ni^2+^ ions by modifying CNFs with a copolymer, methacrylic acid-*co*-maleic acid, via radical polymerization (Figure 19c). The modified aerogel had almost 15 times more carboxyl content than the plain NFC aerogel (7.5 versus 0.54 mmol g^–1^), and the maximum adsorption capacities for the modified aerogel were 165, 135, 138, and 117 mg g^–1^ for Pb^2+^, Cd^2+^, Zn^2+^, and Ni^2+^, respectively. Similar findings were reported by Jin et al.,^241^ who showed that grafting of poly(ethylenimine) (PEI) increased Cu and Pb removal by bacterial cellulose from 90.91 to 111.11 mg g^–1^ and 100 to 125 mg g^–1^, respectively.

Amine-grafted cellulose has also been applied for dye removal by grafting of poly(vinylamine) (PVA)^241^ and PEI^242,243^ to CNFs. PVA-modified CNF aerogels showed high removal capacities for Congo Red (869.1 mg g^–1^), Acid Red (1469.7 mg g^–1^), and Reactive Light Yellow (1250 mg g^–1^).^241^

Sulfhydryl moieties can be grafted into cellulose by carbodiimide coupling of amine groups of cysteine and carboxylate groups of TEMPO-oxidized CNF.^244^ The concentration of grafted thiol ranged from 0.4 ± 0.05 mmol g^–1^ when the ratio of carboxylate to cysteine was 1:2, to 0.9 ± 0.1 mmol g^–1^ when the ratio was 1:8. Thus, increasing the cysteine concentration by a factor of 4 led to a doubling of the thiol concentration. Deposition of these modified CNF onto polyacrylonitrile electrospun fibers resulted in increased adsorption of Cr(VI) and Pb(II) ions (Figure 19d). However, in line with the HSAB theory, higher Cr(VI) adsorption (358 mg g^–1^) was reported for amine-modified PEI-grafted CNCs.^245^

Although aldehyde groups can be 50–150 times fewer than hydroxyl groups on the cellulose surface, i.e., 0.018–0.0738 mmol g^–1^ versus 0.955–2.646 mmol g^–1^, respectively,^160^ carboxyl,^246,247^ thiol,^240,248^ and amino^249^ groups can be grafted to these reducing ends to create adsorbents. Zoppe et al.^249^ attached cationic (*N*-isopropylacrylamide and [2-(methacryloyloxy)ethyl] trimethylammonium chloride) and an anionic polymer (sodium 4-vinyl- benzenesulfonate) to CNCs in aqueous media, demonstrating a protocol for the use of reducing end-modified CNCs that could be applicable in the design of adsorbents for metal ions and dyes.^249^ Thiol and amino-modified CNCs were synthesized by Li et al.^240^ by grafting triazole moieties to end-modified CNCs. While these were used to improve the mechanical properties of natural rubber, the presence of thiol and amino moieties on the CNCs makes the material suitable for complexation of both soft and hard base metal ion pollutants. Zhong et al.^250^ have also recently demonstrated the antimicrobial properties of CNCs modified with thiol groups on their reducing ends, which allowed for templated nucleation of silver nanoparticles.

Grafting of polymers on surface OH moieties and reducing ends of cellulose therefore both present opportunities for increased contaminant removal and water treatment efficacy. We note, however, that the latter presents a challenge with adsorption efficiency due to the lower number of sites available for grafting. Challenges have also been found with characterization, e.g., low signal intensities for NMR, due to the low number of attached groups.^249^

### 5.2 Nanocellulose as Support Materials

This section looks at the application of cellulose as a scaffolding/support material in adsorbents rather than as the active material in process of adsorption itself. The premise for the application of nanocellulose in this manner is simply to take advantage of properties inherent to the material itself. This means that the materials lack the active adsorbent and therefore develop composites with superior porosity, mechanical strength, or increased surface charges.^251−255^ Nanocellulose has also been used to control aggregation^256^ as well as to tune the porosity of aerogels.^257^ Conducting polymers such as poly(pyrrole), polythiophene, and polyaniline are attractive for water treatment due to their high adsorption capacities for metal ions and the ease of synthesis and functionalization.^258,259^ However, they tend to aggregate during synthesis, reducing sorptive surface areas. On its own, poly(pyrrole) has a specific surface area of only 12.21 m^2^·g^–1^ and an adsorption capacity for Cr(VI) of ∼16 mg·g^–1^ at pH 2.^260^ However, when complexed with bacterial cellulose, a specific surface area of 95.9 m^2^ g^–1^ has been reported and the adsorption capacity at the same pH increased to 555.6 mg g^–1^.^261^ Complexation with nanocellulose was also shown to facilitate the use and recovery of polyaniline, which is an excellent adsorbent but whose recovery from treated wastewater is impeded by high buoyancy.^262^

In composites, nanocellulose surfaces also serve as nucleation and growth sites for metal organic frameworks (MOFs) and nanoparticles,^256,263^ as reductants,^264^ or in the control of aggregation.^265,266^ Finally, during adsorption, nanocelluloses can also enhance performance and longevity of adsorbents by controlling attrition/loss of nanoparticles from the adsorbent surface.^267−270^ In the following sections, we survey in detail, nanocellulose-containing composites and their water treatment applications.

#### 5.2.1 Nanocellulose–MOF Composites

Metal organic frameworks (MOFs) are porous crystalline materials made by the interaction of inorganic metal ions or metal clusters and organic ligands.^271^ They are characterized by an infinite network structure, high specific surface area, and thermal stability, in addition to chemical properties unique to the organic and inorganic components.^272,273^ As such, it is possible to tailor MOFs for wide-ranging applications including in water treatment, where they have been used for the adsorption of cations, dyes, and other organic contaminants. However, MOFs come in powders that compact during water flow-though, which not only diminishes flux through the adsorbent column but also limits accessibility of the MOF’s sorption sites, thereby limiting contaminant capture, desorption, and reuse.

Various properties of nanocellulose, particularly the scaffolding ability of CNFs, can address this shortcoming. Furthermore, Zhu et al.^270^ found that in addition to acting as scaffolds for ZIF-8 MOFs, TEMPO-oxidized CNFs also provide nucleation and growth sites for the MOFs. Ionic interactions between Zn^2+^ ions and CNF carboxylic groups led to nucleation and growth of the ZIF-8 crystal. In addition, depletion of MOFs from the aerogel was impeded by hydrogen bonding and ionic interactions between MOFs and CNFs. The rhodamine B adsorption rate (0.036 g mg^–1^ h^–1^) and maximum adsorption capacity (83.3 mg g^–1^) of the aerogels was also much higher than that of plain ZIF-8 MOFs (0.02 g mg^–1^ h^–1^, and 16.81 mg g^–1^). Clearly, therefore, the formation of the CNF–MOF complex significantly improved both the performance and durability of the adsorbent.

Nanocellulose has also been key in the improvement of advanced oxidation processes (AOPs) for water treatment, which involve the use of powerful oxidizing agents, particularly hydroxyl radicals (•OH) and sulfate radicals (SO_4_•−), to degrade organic pollutants. Sulfate radical based AOPs are preferred due to their higher redox potential (2.5–3.1 V) and their applicability over a wider pH range (2–8), hence the increased use of peroxymonosulfate (PMS) as a sulfate radical source. A catalyst is required for the generation of the free radicals and transition metals have traditionally been used for activation of PMS. However, leaching of such elements as Co^2+^ into treated water is an undesirable secondary effect of the process. MOFs can be used to create heterogeneous catalysts, thereby addressing this challenge. Ren et al.^267^ showed that the degradation of *p*-nitrophenol by cobalt containing MOFs was high and rapid (90% of *p*-nitrophenol in 1 h) and that Co ^2+^ loss was reduced. When a zinc-based MOF (ZIF-8) was incorporated into TEMPO-oxidized cellulose nanofibers, good flux (84 L m^–2^ h^–1^ bar^–1^ for 24 h) in a dead-end filtration setup, and high removal of Janus Green B and Methylene Blue dyes (98.9 and 93.8%, respectively) was reported.^274^ Together, these data demonstrate that combining nanocellulose with aerogels can reduce catalyst loss and improve flux by reducing compaction.

**Figure 20:**
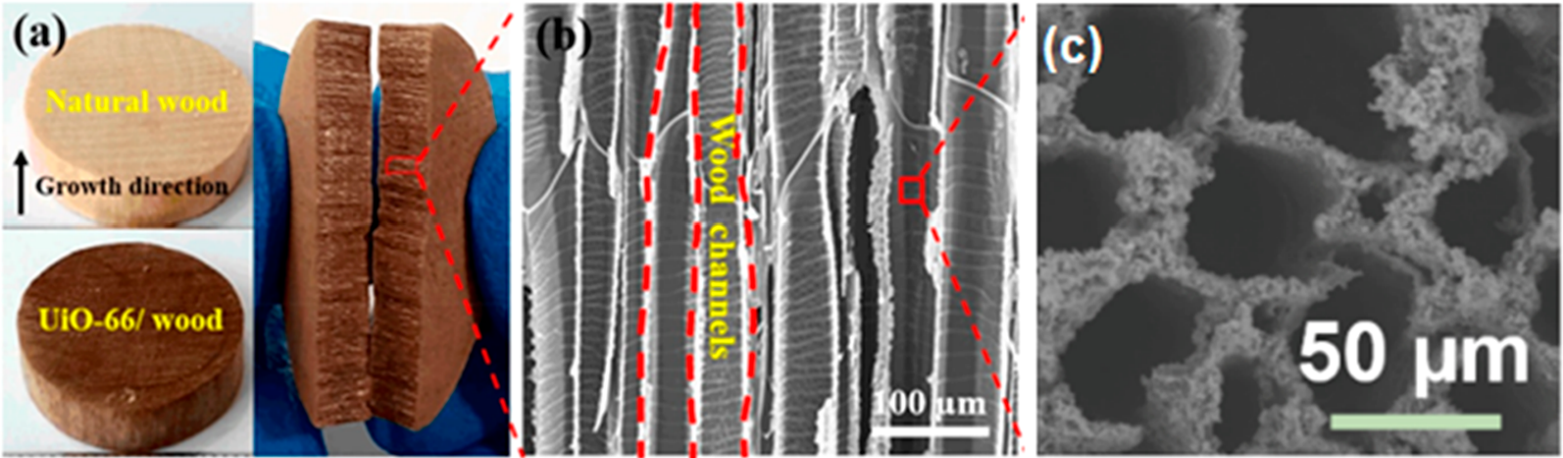
(a) Photographs of natural basswood before (light brown) and after (bark brown) UiO-66/wood deposition,^268^ (b) Scanning electron micrograph of the UiO-66/wood membrane showing the microchannels within the modified wood. Reproduced with permission from ref (268). Copyright 2019 American Chemical Society. (c) SEM image showing polydopamine-bonded MOF nanoparticles anchored on wood microchannels. Reproduced with permission from ref (269). Copyright 2021 Elsevier BV.

There are increasing reports on the use of wood as substrates for MOFs. Wood offers linear channels useful for confinement of MOFs, thus minimizing loss and maximizing remediation efficiency by increasing adsorbent–contaminant contact. Guo et al.^268^ reported the synthesis of a wood–MOF composite by in situ growth of zirconium MOFs in natural basswood (Figure 20). Zirconia MOFs (UiO-66) were formed in the wood channels after adsorption of Zr^4+^ and nucleation on the hydroxyl groups of cellulose. The resulting UiO-66/wood membrane reduced concentrations of rhodamine 6G from 10 to 0096 mg L^–1^ within 5 min by chemical sorption. Furthermore, the removal efficiency, which was much higher than that of activated carbon (60%), remained above 90% after 6 cycles. The membrane was equally effective at removal of the endocrine disruptors, bisphenol A and bisphenol S, 1-naphthyl amine (carcinogen), and propranolol (an antihypertension drug).

Wood–MOF composites have also been used for the capture of metal ions from water. In a strategy that used polydopamine to attain a high MOF loading of zirconium MOFs (UiO-66-NH_2_), Liu et al. attained a 5.81 mg g^–1^ (71.7 wt %) uranyl ion capture from seawater.^269^ Further, selectivity for the ions was maintained even after 30 days in seawater.

A Zn(II)-based MOF magnetic aerogel, however, showed better metal adsorption efficiency, i.e., 558.66 mg g^–1^ for Pb,^275^ and the same could be said for an amine-functionalized Fe-based MOF deployed for reduction of hexavalent chromium under visible light. Cr(VI) reduction capacity was preserved above 80% even after 10 runs.^276^ A note for magnetic adsorbents: the choice of iron precursor can influence the nature of the adsorbent, especially when bacterial nanocellulose is used. While composites made with Fe(II) sulfate and Fe(III) chloride result in higher magnetization than those made with Fe(II) acetate and Fe(III) chloride, the latter produce more porous materials, making them preferable for synthesis of adsorbents.^277^ Together, these studies show that when paired with MOFs, nanocellulose can act as a adsorbent support as well as a template for nucleation and growth of MOFs.

#### 5.2.2 Nanocellulose–Clay Composites

As a result of their high specific surface areas, excellent physical and chemical stability, high cation exchange capacity, and low cost, clays hold great potential as adsorbents of dyes, metal ions, pesticides, and organic matter.^278^ However, just as with MOFs, their powder state substantially hinders applications in water treatment applications. Synergistic effects have, however, been observed for nanocellulose-clay composites, suggesting that in addition to acting as a scaffold, CNF hydroxyl and carboxylate moieties contribute to contaminant sorption. The adsorption of methylene blue, for example, was highest for CNC-montmorillonite composites (183.8 mg g^–1^), than when the clay or CNCs were applied separately i.e., 140.6 mg g^–1^ versus 96.8 mg g^–1^, respectively.^279^

One of the main challenges facing nanocellulose–clay composites is achieving high clay loadings while maintaining their dispersion. Increasing clay concentrations in composites may not always result in higher adsorption capacities. The Cu^2+^ adsorption capacity of CNF–attapulgite complexes decreased from 87.1 to 56.5 mg g^–1^ when the CNF to clay ratio rose from 4 to 12 due to aggregation of the clays and poor pore accessibility at higher clay concentrations.^280^

**Figure 21:**
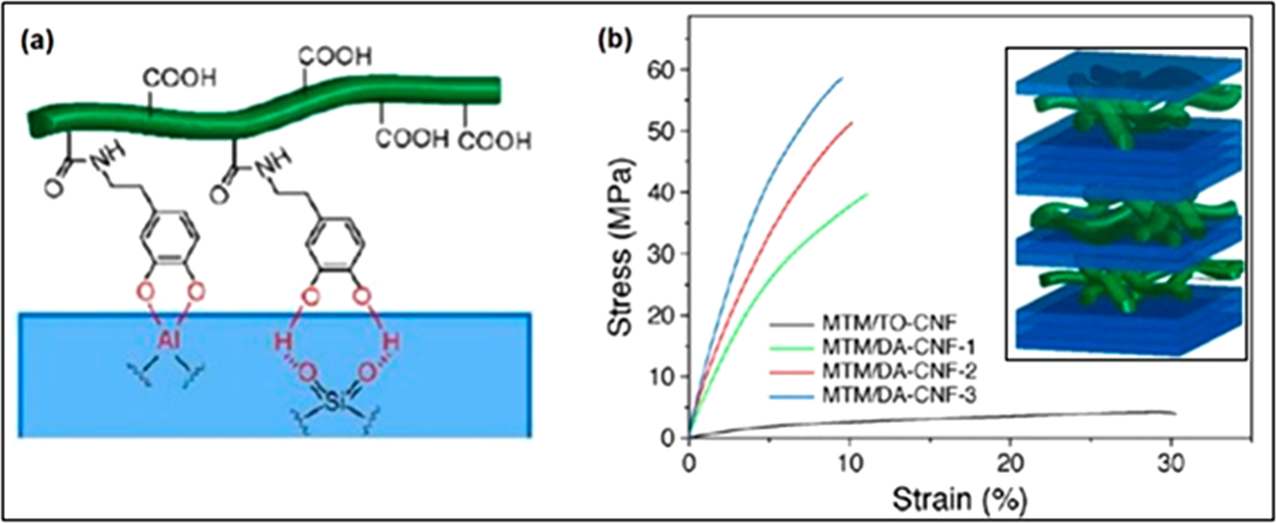
(a) Cross-linking of montmorillonite sheets and cellulose nanofibrils by polydopamine. (b) Stress–strain curves for MTM/TO–CNF and MTM/DA–CNF nanocomposites after swelling in water for 2 h. Inset: Schematic of MTM–CNF composites. Reproduced with permission from ref (281). Copyright 2017 American Chemical Society.

A second challenge concerns the high swelling capacity of clays, which while valuable for water treatment applications because it allows for contaminants to access sorption sites between the clay layers can also pose challenges with the mechanical integrity of adsorbents. Cracks during dehydration can result in the disintegration and mechanical failure of clay composites. Yao et al.^281^ reported a technique to enhance moisture resistance in CNF–montmorillonite composites that could also be useful for adsorbents in water treatment applications. In their work, dopamine functionalized CNFs were conjugated to montmorillonite (MTM) platelets via catechol/metal ion chelation and hydrogen bonding to generate layered nanocomposite films (Figure 21a). When the films were soaked in water, composites with unfunctionalized CNF, i.e., MTM/TO–CNF, increased in thickness from 33 to 293 μm in 1 min and had a water absorbance of 580% due to the high hydrophilicity of both MTM and CNFs. In contrast, films made with dopamine-functionalized CNFs only increased from 35 to 112 μm after 2 h and had a saturation value of 179%. Hydrophobicity imparted by polydopamine, and covalent bonding between MTM sheets and the polydopamine-functionalized CNFs, imparted stability to the composites. Further, even in their hydrated form (water content of 64 wt %), composites with polydopamine-functionalized CNFs had a tensile strength of 57.4 ± 2.2 MPa (Figure 21b), which is much higher than conventional polymer hydrogels.

### 5.3 Nanocellulose in Membrane Filtration

Cellulose, particularly in its acetate form, has been applied in membrane filtration for decades, with the membranes functioning largely as carriers of adsorbents, e.g., clays, graphene oxides, MOFs, carbon nanotubes, and other nanomaterials, or catalysts for dye and organics degradation. As these have been widely discussed in various reviews,^282−285^ we restrict our discussion here to nanocellulose, the action as active adsorbent materials, and their influence on membrane performance during water treatment.

Nanofiltration membranes (1–10 nm pore diameters) are attractive in water treatment because of their higher flux and lower operating pressure and energy consumption compared to reverse osmosis membranes. Thin film composite membranes, which are commonly used in nanofiltration, are usually fabricated by the interfacial polymerization of piperazine and trimesoyl chloride on an ultrafiltration substrate. However, as with other polymeric membranes, thin film composite membranes are challenged by chorine degradation, biofouling, and flux reduction, etc. While nanomaterials including zeolites, graphene oxides, and carbon nanotubes have been used to address these challenges, poor compatibility with these inorganic materials and the polyamide skin layer often results in their detachment or dissolution from the polyamide layer.^286^ Incorporating CNCs in the polyamide layer has, however, been shown to result in composite membranes with increased hydrophilicity (contact angle decreased from 60° to 38°) and an increased water flux (78.9 to 106.9 L·m^–2^ h^–1^) without nanoparticle loss.^287^ The membranes were also resistant to chlorine degradation even after a 4 h exposure period to a 6000 ppm chlorine solution. Good chlorine resistance and flux enhancement were also reported for membranes with TEMPO-oxidized CNFs.^288^

A separate approach involves incorporation of nanocellulose as interlayers between the hydrophobic ultrafiltration membrane and the functional thin film layer. Besides improving permeability, these interlayers improve the coating efficiency of the substrate without sacrificing salt rejection.^289^ In mixed matrix membranes, nanocelluloses also increase hydrophilicity, water flux (92–195 L·m^–2^ h^–1^), and bacterial resistance.^155^ They can be incorporated into membranes during, or after, synthesis. Postsynthetic modification by carbodiimide conjugation of thiol-modified CNFs to an electrospun polyacrylonitrile membrane resulted in a high flux microfiltration membrane with Cr(VI) and Pb(II) adsorption capacity of 87.5 and 137.7 mg g^–1^, respectively.^244^ The synthetic approach, however, is lengthy. Nevertheless, these membranes had a higher sorption efficiency for Pb(II) than that of thiol-modified membranes prepared by deacetylation of electrospun cellulose acetate (22 mg g^–1^).^290^

To prepare nanocellulose-modified electrospun membranes in a single-step process, water-in-oil emulsions are used to stabilize nanocellulose suspensions in the polymer dope. During spinning, the solvent in the region close to the surface evaporates faster, resulting in higher viscosity in the outer layer and inward movement of emulsion droplets to the center, where they are simultaneously condensed and stretched under the force of a high voltage field.^291^ Manipulation of the humidity conditions can allow for the production of porous fibers,^292^ which could expose the enclosed CNCs and modify the surface and adsorption properties of the electrospun membrane.

### 5.4 Nanocellulose Absorbents in Oil–Water Separation

Besides large-scale marine oil spills from shipping and oil exploration, many industries including metal/steel industries and mining, produce oily wastewater. A typical mining operation, for example, loses over one-fifth of its total oil purchases through spills, leaks, and overflows, producing 140000 L of oil-contaminated water each day.^293^ There is therefore a need for absorbents for treatment of oil-contaminated water. Nanocellulose is attractive as a material for the design of absorbents for oil/water separation because of its ability to form light aerogels with high surface areas that can be hydrophobically modified to increase oleophilicity and hydrophobicity. Although these have been mentioned a number of times already, aerogels from nanocellulose may be synthesized via chemical vapor deposition,^157,294−296^ click chemistry,^297−299^ and atomic layer deposition.^300^

**Figure 22:**
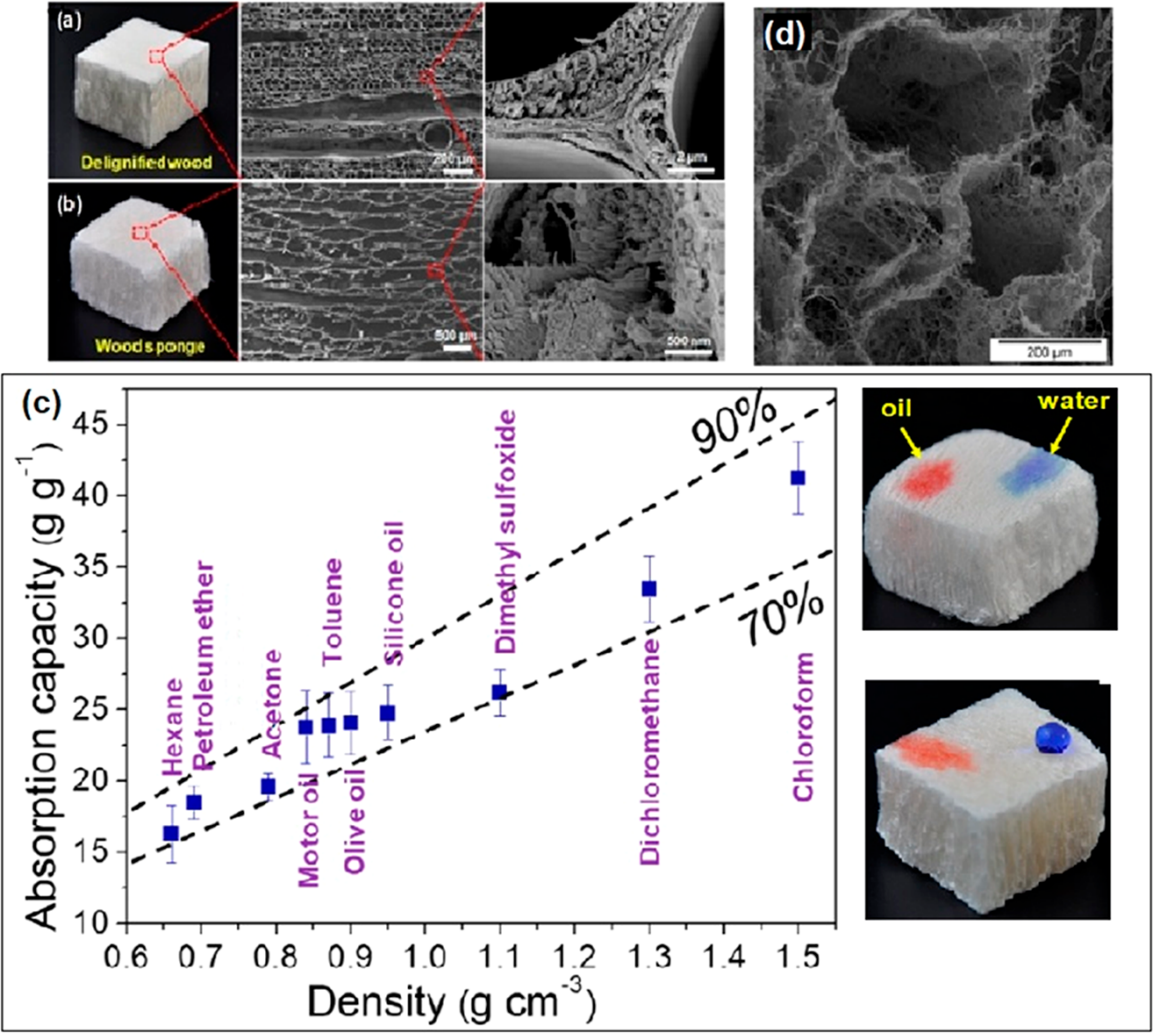
(a) Photograph and scanning electron microscopy (SEM) images showing cross-sectional view of the honeycomb structure of delignified wood. (b) Photograph and SEM image of cross-sectional view of the wood sponge showing the lamella structure and exposed cellulose nanofibers in cell wall. (c) Oil absorption performance of silylated wood sponge and photographs of water and oil droplets on pristine wood sponge (top) and silylated wood sponge (bottom), chloroform, and toluene. (c) Representative curves of repeated cycles for diesel oil and diesel/water mixtures. Reproduced with permission from ref (301). Copyright 2018 American Chemical Society. (d) SEM image of aerogel made using 0.4 wt % aqueous solution of electrospun cellulose fibers. Reproduced with permission from ref (302) (CC-BY).

Typically, the use of absorbents for oil/water separation entails mechanical forces to expel absorbed oil between absorption cycles. Mechanical stability and compression recovery are, therefore, essential for a good absorbent. This has made delignified wood, e.g., balsa wood (*Ochroma pyramidale*), an attractive template for the design of adsorbents. The process involves delignification to produce sponges with a honeycomb^295^ or a lamella structure.^301^ The latter seems to have superior compression recovery than the former. Lamella wood sponges can bear compressive strain as high as 60% and fully recover to their original height after the stress is removed: a height retention of 93% after 100 compression cycles. Honeycomb-structured sponges, in contrast, only recovered to 60% of their original height. This suggests that the additional treatment step, namely using NaOH to break down the honeycomb structure (Figure 22a) to a spring-like lamella one (Figure 22b) contributes to its mechanical robustness.

Treatment of the lamella-structured sponge with methyltrimethoxysilane made it hydrophobic but preserved its structure. Its oleophilic properties were evidenced by its high uptake capacity, 16–41 times its own weight, of a wide range of oils, e.g., motor oil, silicone oil, and organic solvents, e.g., hexane and chloroform (Figure 22c).

Despite lower compressive moduli, high absorption capacities have been reported for nanocellulose–aerogel composites. A CNF/natural rubber latex composite was found to have a 92% porosity and a surface area of 226 m^2^ g^–1^. Sorption capacities ranging from 20 to 50 g g^–1^ in 3 s for soybean and sunflower oil, as well as diesel and gasoline, were reported.^294^ CNFs also modulated porosity of the natural rubber composite so that when its content was too low, no internal walls were observed, and the foam was fragile. Increasing the amount of CNF resulted in foams with greater interconnectivity, larger pores (∼250 μm), and larger surface areas, while those with lower CNF content had compact structures with smaller pores sizes (∼50 μm). CNFs also acted as anchors for the NR latex particles responsible for hydrophobicity.

An interesting iteration to the adsorption by cellulose-derived nanoscale fibers has been recent reports of the use of electrospun fibers to generate aerogels. Electrospun cellulose acetate fibers treated by alkaline hydrolysis were disintegrated into smaller fragments and freeze-dried to generate aerogels.^302^ The high absorption capacity of the aerogels 373 g g^–1^ was attributed to the interconnected pore structure and the high surface area provided by the fibrous cellular walls (Figure 22d). The aerogel’s absorption of chloroform attained 95% of the theoretical absorption capacity. Furthermore, the aerogels had 89% shape recovery after 70% compressive strain, suggesting that they could quickly and substantially recover from repeated squeezing during absorption.^302^

Polyvinyl acetate (PVA)–cellulose aerogels, on the other hand were found to be compressible for 50 cycles without any significant decrease in mechanical strength and with an absorption capacity up to 32.7 times of their weight when absorbing chloroform from water.^303^ These hydrophobic aerogels were prepared by thermal chemical vapor deposition of methyltrichlorosilane on the surface of PVA/CNF. Zheng et al.^157^ showed that after silane coating, aerogels completely recovered their original shape with no mechanical failure after being subjected to 80% strain. This contrasted with noncoated aerogels, which were deformed permanently when the compression strain was more than 20%. The maximal stress at 80% strain normalized by the density was found to be 3.8 ± 0.2 MPa cm^3^ g^–1^. Interestingly, the aerogels were capable of adsorbing metal ions from water (Hg(II), Pb(II), Cu(II), and Ag(I)), implying that they maintained surface charges enough for electrostatic adsorption of the ions, even after the hydrophobic treatment.

### 5.5 Nanocellulose in Water Disinfection

Uncharged nanocellulose has no intrinsic antimicrobial properties. However, oxidized variants, e.g., by 2,3-dialdehyde nanocellulose, have potent and often immediate antimicrobial action in vivo and in vitro.^304^ The low pH they exert has negative effects on protein activity, membrane permeability, and nutrient absorption.^305^ However, an acid-dependent strategy would be limited in water treatment applications due to dilution and neutralization.

Notwithstanding, several nanocellulose antimicrobial materials suitable for water treatment have been synthesized by (i) surface modification with polymers having antimicrobial properties, e.g., quaternary ammonium compounds,^194,306−308^ or (ii) complexation with nanomaterials having antimicrobial properties including zeolites,^309^ graphene oxide,^154^ organic agents,^310^ and metal/metal oxide nanoparticles, e.g., Ag,^264,311^ ZnO,^312^ and TiO_2_.^313^ In this latter approach, nanocelluloses may act as nanoparticle nucleation sites to decrease nanoparticle agglomeration, thereby increasing sorptive surface areas and antimicrobial action,^265^ or they may act as reductants.^311^ Taking advantage of the reductive properties of TEMPO-oxidized CNF, Zhang et al. prepared Ag-doped nanofibers by hydrothermal synthesis.^264^ The resulting composite had antimicrobial action toward both Gram negative (*Escherichia coli*) and Gram positive (*Staphylococcus aureus*) bacteria, as well as a strain of fungus (*Candida albicans*). Nath et al.^266^ also reported antimicrobial action by a polyaniline supported ZnO/CeO_2_/nanocellulose composite against *E. coli* and *Bacillus subtilis*, in addition to a high adsorption efficiency for arsenic ions. In their work, the presence of nanocellulose inhibited the aggregation of the ZnO/CeO_2_ nanoparticles, thereby promoting an even incorporation in the polyaniline matrix.

The other strategy for generating nanocellulose with antimicrobial properties involves grafting of polymers, e.g., quaternary ammonium compounds (QACs), which possess these properties. Positive centers in QACs bind to the negatively charged phosphate groups in peptidoglycans of Gram-positive bacteria or phospholipids of Gram-negative bacteria. The bacterial cell membranes are gradually disrupted, leading to membrane rupture and cell death.^314^

**Figure 23:**
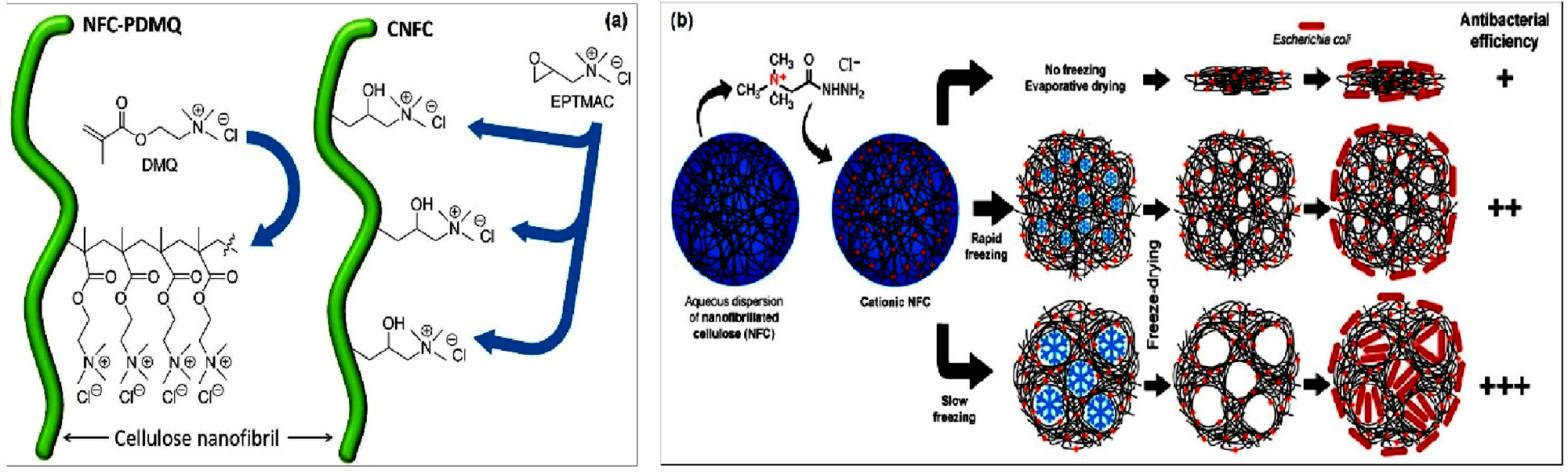
(a) Nanocellulose cationisation by grafting of [2-(methacryloyloxy)ethyl]trimethylammonium chloride (DMQ) and etherification with EPTMAC. Reproduced with permission from ref (306) (CC-BY). (b) Schematic of the effects of rapid and slow freezing in pore structure of aerogels and the effect of this on their antibacterial properties. Reproduced with permission from ref (194) (CC-BY).

QACs can be introduced onto nanocellulose using different approaches. Littunen et al.^306^ compared cationization of CNFs by etherification with epoxypropyl trimethylammonium chloride (EPTMAC) and by redox-initiated graft copolymerization using [2-(methacryloyloxy)ethyl] trimethylammonium chloride (Figure 23a). They found that while materials from both techniques had a broad spectrum of antimicrobial activity, etherification resulted in a higher degree of substitution and charge density. As a result, CNFs produced by etherification had higher antimicrobial activity, particularly for Gram negative bacteria, than those produced by copolymerization. Otoni et al.^194^ showed pore size and accessibility of inner surfaces of aerogels as key to optimal antimicrobial activity (Figure 23b). It can be inferred that close packing of quaternary ammonium groups in copolymerized CNFs diminished their antibacterial effect due to poor access to sorption sites located inside the aerogel. Adsorption of anionic contaminants onto the QAC-modified surfaces, however, would be expected to result in a neutrally charged surface. Second, there is evidence suggesting reduced inhibitory effects of some QACs and, as a result, bacterial resistance after long-term exposure.^315^ These two issues present areas for further research.

### 5.6 Nanocellulose in Solar Desalination

For areas without well-established water and energy infrastructure, thermal desalination by solar energy is a potential route to low-cost, modular, and potable drinking water because these desalinators can be fabricated with readily available low-cost materials and do not require electricity. Solar thermal desalination works by using solar radiation to heat up saline or other contaminated water. The vapor generated is then condensed, producing clean water, with up to 1.83 L m^–2^ h^–1^ of water being produced from such systems.^316,317^

**Figure 24:**
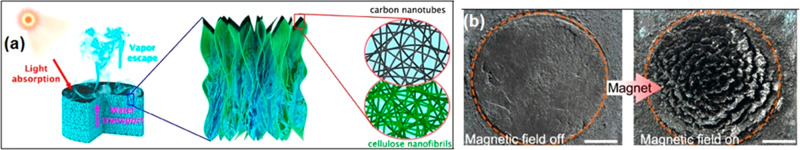
(a) Bilayered solar absorber aerogel comprising carbon nanotubes atop cellulose nanofibers. Reproduced with permission from ref (318). Copyright 2018 American Chemical Society. (b) Photograph of cellulose–magnetite mixtures for solar absorption, before and after magnetic stirring (scale bar: 1 cm). Reproduced with permission from ref (320). Copyright 2021 American Chemical Society.

Cellulose nanofibers are particularly amenable to fabrication of solar desalinators due to their hydrophilicity, which favors water transport, high aspect ratio, which allows for channel formation upon directional freezing, and good mechanical properties.^316−319^ To optimize radiation absorption, materials, e.g., carbon nanotubes (CNT),^318^ TiO_2_^319^ have been used as solar absorbers atop bacterial or plant cellulose (Figure 24a). When exposed to solar radiation at 1.1 kW m^–2^, the CNT-coated aerogel absorbed 97.5% of incident light from 300 to 1200 nm, resulting in an evaporation rate of 1 L m^–2^ h^–1^. A similar water production rate: 1.26 L m^–2^ h^–1^ was reported for a black titania/bacterial cellulose solar absorber.^319^

Gan et al.^320^ attempted to improve the solar absorption efficiency of magnetite by increasing adsorptive surface areas. By magnetically stirring cellulose–magnetite mixtures, the absorbents were transformed from 2-dimensional planar structures with 76.9% solar absorption to 3-dimensional structures (Figure 24b) with 90.6% absorption under 1 sun illumination. The evaporation rate also increased from 1.19 to 1.39 L m^–2^ h^–1^. However, salt deposition on the 3D cellulose film slightly decreased performance after 6 h.

Zou et al.^317^ attempted to address this challenge of salt deposition by coating the cellulose aerogels with polydopamine. The resulting material had, in addition to resistance to salt deposition, greater antifouling capacity due to increased surface roughness, high hydrophilicity, and dye sorption capacity for methylene blue and rhodamine B. Furthermore, surface temperatures could rise from 22 to 40 °C in 10 min, with 1 sun radiation in both seawater and oil-contaminated water. The superhydrophilic PDA-coated cellulose aerogels repelled the oil contaminants and delivered water to the surface of the aerogel, resulting in an evaporation rate of 1.36 L m^–2^ h^–1^. No salt deposits were found on the aerogel after 10 days, and the evaporation rate was not diminished, confirming the durability of the aerogel.

Resistance to disintegration in the harsh conditions of saline water poses a significant challenge for aerogel use in thermal solar desalination. Silane functionalization seems to hold some potential in addressing this challenge by imbuing aerogels with superhydrophilicity and interconnected radial-aligned channels that allow for high water transfer rates from solution to the aerogel surface.^316^ A water evaporation efficiency of 95.9% and an evaporation rate of 1.83 L m^–2^ h^–1^ was reported when these aerogels were covered with a layer of soot. When used to generate clean water from an oil–water mixture, the absorption capacity decreased only slightly, from 34 to 29 g g^–1^, even after 29 adsorption–removal cycles and a stable evaporation rate of 1.75–1.83 L m^–2^ h^–1^ while irradiated continually for 10 days, suggesting it could withstand long-term deployment. Nevertheless, as with all treatment technologies, water production rate, cost, and durability will be key for determining market entry for solar desalination devices. It is, nevertheless, cheaper than bottled water but more expensive than tap water, and as there are still locations around the world where tap water is unavailable, solar desalinators may be worth considering in such situations.

## 6 Conclusions

Cellulose has a unique interaction with water. This interaction is something which needs to be harnessed in the drive toward green and sustainable chemistries for a range of applications, where the replacement of oil-based materials is of paramount importance and need. In the cell wall, cellulose intimately contacts with other materials, and water plays a key role in mediating those interactions in what perhaps could be considered the most green of manufacturing processes. When extracted from the plant cell wall, cellulose continues to interact with water, something which can be exploited in the self-assembly of higher-order structures. This review has covered those, including chiral nematic liquid crystals and amphiphilic cellulose derivatives. More research is needed in these areas to better understand how water mediates these self-assembly processes. This will be key in our movement toward a green materials revolution, with cellulose at the center of this to produce new technologies. As human beings we also face grave difficulties in accessing clean water, particularly as climate change takes effect. In water treatment applications, nanocellulose presents benefits, ranging from strengthening and increasing flux and antifouling efficiency of membranes to applications in solar desalination, and as hosts of reactive agents including catalysts, clays, and other nanoparticles. While many studies have investigated the optimization of contaminant capture by these materials, including applications to real samples, consideration needs to be given to other water components, particularly organic matter, to obtain accurate efficiencies of adsorbent materials.

For low-cost treatment solutions, nanocellulose is an expensive option largely due to the production methods. However, as shown by some of the studies reviewed, the performance of less processed microfibrillated cellulose can sometimes be comparable to that of nanofibrillated alternatives. More studies comparing the performance of these two sets of materials are needed to determine instances when nanoscale or micrometer-scale are most appropriate.

Large-scale application of nanocellulose in water treatment also calls for synthetic approaches that are greener and leaner, economically. Deep eutectic solvents are one route through which materials can be synthesized using reagents that are relatively cheap, biodegradable, and environmentally friendly. Mechanical treatment in the absence of heat and solvents may be another alternative.

## Data Availability

This study did not involve any underlying data.
